# High Prevalence of Coinfecting Enteropathogens in Suspected Rotavirus Vaccine Breakthrough Cases

**DOI:** 10.1128/JCM.01236-21

**Published:** 2021-11-18

**Authors:** Ceren Simsek, Mandy Bloemen, Daan Jansen, Leen Beller, Patrick Descheemaeker, Marijke Reynders, Marc Van Ranst, Jelle Matthijnssens

**Affiliations:** a KU Leuven—University of Leuven, Department of Microbiology, Immunology and Transplantation, Rega Institute for Medical Research, Leuven, Belgium; b Department of Laboratory Medicine, Medical Microbiology, AZ Sint-Jan Brugge-Oostende AV, Bruges, Belgium; University of Iowa College of Medicine

**Keywords:** enteric coinfections, gastroenteritis, NGS, RT-qPCR, vaccine breakthrough, rotavirus

## Abstract

Despite the global use of rotavirus vaccines, vaccine breakthrough cases remain a pediatric health problem. In this study, we investigated suspected rotavirus vaccine breakthrough cases using next-generation sequencing (NGS)-based viral metagenomics (*n* = 102) and a panel of semiquantitative reverse transcription-PCR (RT-qPCR) (*n* = 92) targeting known enteric pathogens. Overall, we identified coinfections in 80% of the cases. Enteropathogens such as adenovirus (32%), enterovirus (15%), diarrheagenic Escherichia coli (1 to 14%), astrovirus (10%), *Blastocystis* spp. (10%), parechovirus (9%), norovirus (9%), Clostridioides (formerly *Clostridium*) difficile (9%), Dientamoeba fragilis (9%), sapovirus (8%), Campylobacter jejuni (4%), and Giardia lamblia (4%) were detected. Except for a few reassortant rotavirus strains, unusual genotypes or genotype combinations were not present. However, in addition to well-known enteric viruses, divergent variants of enteroviruses and nonclassic astroviruses were identified using NGS. We estimated that in 31.5% of the patients, rotavirus was likely not the cause of gastroenteritis, and in 14.1% of the patients, it contributed together with another pathogen(s) to disease. The remaining 54.4% of the patients likely had a true vaccine breakthrough infection. The high prevalence of alternative enteropathogens in the suspected rotavirus vaccine breakthrough cases suggests that gastroenteritis is often the result of a coinfection and that rotavirus vaccine effectiveness might be underestimated in clinical and epidemiological studies.

## INTRODUCTION

Group A rotaviruses (referred to here as rotavirus) can cause gastroenteritis in infants less than 5 years old, leading to high morbidity in developed settings (1). The rotavirus genome consists of 11 double-stranded RNA (dsRNA) segments encoding 6 structural proteins (VP1 to VP4, VP6, and VP7) and 6 nonstructural proteins (NSP1 to NSP6). The rotavirus outer capsid proteins, VP7 and VP4, form the basis for a dual classification into G and P genotypes, respectively (2). To account for genomic evolution, this dual classification has been extended to all 11 genes, establishing so-called genotype constellations (2–4). Two of them, Wa-like and DS-1-like, are responsible for most human infections and are designated I1-R1-C1-M1-A1-N1-T1-E1-H1 and I2-R2-C2-M2-A2-N2-T2-E2-H2, respectively, for the non-G and non-P genotypes (4).

There are two oral live-attenuated vaccines against rotavirus gastroenteritis that are used worldwide: Rotarix (GlaxoSmithKline, Belgium) and RotaTeq (Merck & Co., Inc., USA). They have decreased the rotavirus gastroenteritis burden significantly and are highly effective against rotavirus gastroenteritis in countries with low child mortality (5). Rotarix was introduced in Belgium in 2006 and immediately reached a high degree of coverage. Since then, there has been exhaustive surveillance of the possible effect of vaccine introduction on the rotavirus genotype distribution. It was observed that G1P[8] was dominant in the prevaccine period, whereas G2 became dominant after vaccine introduction, albeit with strong genotype fluctuations in the following years (6). There are also similar postvaccination reports from Brazil, South Korea, and Australia on increased G2 dominance, although a causal link with vaccine introduction is currently lacking (7–10).

Although the vaccines provide heterotypic immunity, it is uncertain whether the protection will extend against completely heterotypic and newly emerging rotavirus strains. Therefore, it is crucial to screen the natural and/or vaccine-induced changes in the circulating rotavirus population. On the other hand, a third of the yearly rotavirus infections are asymptomatic in children younger than 2 years old (11), and there is little screening for most other enteropathogens which might cause (a)symptomatic infections. Sporadic diarrhea can also be caused by viruses such as noroviruses, enteric adenoviruses (type 40/41), astroviruses, sapoviruses, and picornaviruses; bacteria such as Campylobacter jejuni, enteropathogenic Escherichia coli, *Shigella*, *Yersinia*, Shiga-toxin producing E. coli, and Salmonella; and parasites such as *Cryptosporidium*, Giardia lamblia, and Entamoeba histolytica (12–15). Moreover, enteric coinfections might impact gastroenteritis progression in infants. Previous studies have shown prevalent coinfections, as well as longer diarrhea and increased hospitalization, associated with coinfections in rotavirus gastroenteritis (16–19). We speculate that often, gastroenteritis diagnosed as of rotavirus etiology in vaccinated infants might actually be a coinfection or entirely caused by another pathogen and that rotavirus was present only as an asymptomatic infection.

In light of this, we performed a combination of a nontargeted next-generation sequencing (NGS) and a sensitive reverse transcription-PCR (RT-qPCR) approach to screen for enteric viruses, bacteria, and parasites in suspected Rotarix vaccine breakthrough cases in Belgium. Our first aim was to investigate the rotavirus genotypes and genotype constellations. Secondly, we aimed to investigate enteric coinfections, as well the genetic diversity of the coinfecting viruses. Thirdly, we attempted to identify the most likely cause of gastroenteritis, by estimating the contribution of each pathogen to the disease. Ultimately, we believe that this study will contribute to a better assessment of rotavirus disease burden and rotavirus vaccine effectiveness.

## MATERIALS AND METHODS

### Selection of rotavirus vaccine breakthrough cases.

Rotavirus-positive fecal samples have been collected from patients throughout Belgium as part of Rotavirus Surveillance Network Belgium (RSNB) and the National Reference Center (NRC) activities for rotavirus. Stool samples were initially screened for rotavirus antigen (e.g., enzyme immunoassay [EIA]) or RNA (RT-PCR-based techniques) by hospitals, physicians, or diagnostic centers, and positive samples were sent to the Leuven University Hospital (UZ Leuven). The VP7 and VP4 genes of the rotaviruses are routinely RT-PCR amplified and Sanger sequenced to determine their G and P genotypes, as described previously (6). For the current study, we focused on 915 patients who were known to have received a full Rotarix vaccination regimen (2 doses) between the 2007-2008 and 2017-2018 rotavirus seasons. From these patients, we selected a representative set of 8 to 13 samples per season for sequencing analysis. The final identification of 102 rotavirus vaccine breakthrough cases was made based on the following criteria: (i) there must be at least 15 days between the episode of gastroenteritis and completion of the Rotarix vaccination regimen (https://www.ema.europa.eu/en/medicines/human/EPAR/rotarix), (ii) the selected samples must represent the G and P genotype distribution present in that respective rotavirus season, and (iii) the partial VP7 and VP4 sequences obtained from the samples must not share high nucleotide similarity (>99%) with Rotarix or RotaTeq vaccine strains, as confirmed with BLASTn analysis. The study was approved by the Ethics Committee Research (EC Research) of UZ Leuven with the reference number S64614. Details about the selected 102 samples (year of sample collection, rotavirus genotype, sex, age at sample collection, time between last Rotarix vaccination and sample collection, and NGS reads) can be found in Table S1 in the supplemental material.

### Viral metagenomics.

The NetoVIR protocol was used for viral enrichment of the fecal suspensions as described before (20). Briefly, the fecal samples were suspended in Dulbecco’s phosphate-buffered saline (dPBS) and homogenized with a Minilys homogenizer (Bertin Technologies) for 30 s at 4,000 rpm. The homogenates were centrifuged for 3 min at 17,000 × *g* and filtered with 0.8-μm polyethersulfone (PES) filters (Sartorius). Filtrates were treated with Benzonase (Novagen) and micrococcal nuclease (New England Biolabs) at 37°C for 2 h to remove the free-floating nucleic acids. Subsequently, DNA and RNA were extracted using the QIAamp viral RNA minikit (Qiagen), without addition of carrier RNA. Reverse transcription and second-strand synthesis were performed by an adjusted version of the whole-transcriptome amplification (WTA2; Sigma-Aldrich) protocol as described previously (21). A sequencing library was constructed with the Nextera XT library preparation kit (Illumina). The size of the library was checked with a Bioanalyzer (Agilent Technologies) with a high-sensitivity DNA chip, and the 2 nM pooled libraries were sequenced on either an Illumina NextSeq 500 platform (2 × 150-bp paired-end) or a NovaSeq 6000 platform (2 × 150-bp paired-end).

### NGS data analysis.

Low-quality reads, ambiguous bases, and primer and adapter sequences were removed from the paired-end reads with Trimmomatic v0.36 with default parameters (22). Sequences mapped to the human genome reference (hg38) and negative-control sequences were removed. Quality-controlled reads were *de novo* assembled with metaSPAdes v3.14.0 using 21, 33, 55, and 77 k-mer lengths (23). The contigs were concatenated, subjected to a 600-bp length cutoff, and clustered on 95% nucleotide identity covering 80% of the contig using a perl script (24), to generate a nonredundant contig set. This contig set was annotated with DIAMOND v0.9.10 against a nonredundant protein database (25). The eukaryotic viral contigs were extracted with an in-house script. Where possible, the incomplete viral genomes were completed *in silico* by mapping the quality-controlled reads against the reference sequences determined by the highest BLASTn nucleotide similarity with the lowest E value using BWA software v0.5.9 (26) and SAMtools v1.6 (27). BWA and SAMtools were also implemented to obtain the relative abundances by mapping the quality-controlled reads to the non-redundant contig set. Only the contigs that were covered in length by 70% were kept for further analyses, and the contigs that had fewer than 100 mapped reads were discarded.

### Enteric virus characterization and phylogenetic analyses.

The NGS read counts were normalized to the total quality-controlled reads of the corresponding sample, as well as the genome length of the virus of interest, and log_10_-transformed to obtain relative abundances of contigs that represent viral genomes. In order to generate relative abundance heat maps based on NGS data, Reshape2, tidyverse, and complex heat map R packages were used (28–30). Enteroviruses, noroviruses, and sapoviruses were genotyped with the National Institute for Public Health and the Environment (RIVM) genotyping tool (31). Rotavirus gene segments were genotyped using the Virus Pathogen Database and Analysis Resource (ViPR) (32). The presence of vaccine(-derived) sequences was investigated by mapping quality control (QC) reads per sample to Rotarix (accession numbers KX954616 to KX954624) and RotaTeq (accession numbers GU565041 to GU565051 and GU565063 to GU565095) sequences using BWA and SAMtools. Open reading frames were determined by the NCBI ORF Finder tool (33) (www.ncbi.nlm.nih.gov/orffinder). Only sequences that cover at least 85% of the ORF are included in the phylogenetic analyses. Nucleotide-level multiple sequence alignments were generated using MUSCLE (34) with default parameters in MEGA software v7.0.26 (35), except for adenovirus sequences, where MAFFT v7 is used with auto mode, due to better scalability with longer genomes (36). The nucleotide substitution models were predicted using jModelTest v2.1.10 for all enteric-virus alignments (37). An optimized number of bootstrap replicates (100 to 1,000) was determined by the autoMRE option, and maximum likelihood trees were generated with RAxML-NG v0.9.0 (38). The rotavirus genotype representations next to the VP7 phylogeny in Fig. 3 were generated in R with ggtree package (39). Enteric virus phylogenies and rotavirus VP1 to -3 and NSP2 to -5 gene trees were rooted at the midpoint. Outgroup rooting was performed for VP4 (G8P[14] PR1973 strain), VP7 (G6P[15] Roe deer strain), and NSP1 (G6P1A[8] RotaTeq strain) genes. Accession numbers for all the reference strains are given in Table S7.

### Enteropathogen detection with RT-qPCR.

Fecal dilutions (10%, PBS) of the 92 samples from suspected rotavirus vaccine breakthrough cases, of which sufficient material was left from the initial 102 (Table S1), were transported to the AZ Sint Jan Brugge-Oostende clinical laboratory, mixed with DNAzol buffer (Thermo Fisher), and stored at −80°C until further analysis. Next, RNA/DNA extraction was performed on the QIAsymphony SP (Qiagen), and a RT-qPCR was performed with a customized TaqMan array card targeting gastrointestinal pathogens, in batches of 8 samples. The target genes were detected in 48 uniplex real-time PCRs on a ViiA 7 system (Thermo Fisher). The tested pathogens included 8 viruses: norovirus (genotypes I, II, and IV), adenovirus, astrovirus, sapovirus (genotypes I, II, IV, and V), rotavirus (serogroup A), enterovirus, hepatitis E virus (HEV), and human parechovirus. They also included 10 bacteria: toxigenic Clostridioides (formerly *Clostridium*) difficile, Campylobacter spp. (Campylobacter jejuni, C. coli, C. lari, C. upsaliensis, and C. hyointestinalis), Salmonella sp., enteroaggregative E. coli (EAEC), enteroinvasive E. coli (EIEC), enteropathogenic E. coli (EPEC) (atypical, *eaeA* positive; typical, *eaeA* and *bfpA* positive), enterotoxigenic E. coli (ETEC), Shiga-toxin producing E. coli (STEC), Yersinia enterocolitica, Yersinia pseudotuberculosis. Last, they included 8 parasites: Giardia lamblia (syn. Giardia duodenalis), *Cryptosporidium* sp., *Entamoeba* spp. (E. histolytica), Strongyloides stercoralis, Dientamoeba fragilis, *Blastocystis* spp., *Microsporidium* spp. (Enterocytozoon bieneusi, Encephalitozoon hellem, Encephalitozoon intestinalis, and Encephalitozoon cuniculi), and *Schistosoma* spp. (Schistosoma mansoni, S. intercalatum, S. haematobium, S. guineensis, S. mekongi, and S. japonicum). Phocine distemper virus (PDV) was added during nucleic acid extraction as an experimental control (40). The 18S rRNA gene was used as an internal control. The primer sequences are proprietary to the AZ Sint Jan Brugge-Oostende and can be made available for noncommercial purposes upon request. The RT-qPCR data were interpreted as follows: (i) the quantification cycle (*C_q_*) value, defined as the number of PCR cycles where the fluorescent signal was higher than the detection threshold (41), was determined and (ii) a *C_q_* value greater than or equal to 40 was accepted as negative for all pathogens. Additional information on how the analytical performance of the qPCR assays was determined can be found in the supplemental materials and methods.

*C_q_* values for each standard per sample and the spread of their distribution are shown in Table S2. To assess the gastroenteritis etiology, a score was given for each of the pathogen-specific RT-qPCR assays (including rotavirus): strongly positive, 3 points; positive, 2 points; weakly positive, 1 point. The relationship between the *C_q_* scores and the clinical scoring is shown in Table S6. Subsequently, we calculated the ratio of the sum of alternative pathogen scores present in each sample, over the rotavirus score.

### Data availability.

The data have been deposited with links to BioProject accession number PRJNA729919 in the NCBI BioProject database (https://www.ncbi.nlm.nih.gov/bioproject/). The sequences have been deposited in GenBank, and the accession numbers can be found in Table S8.

## RESULTS

### Sample selection from suspected rotavirus vaccine breakthrough cases.

In this study, we explored coinfecting enteropathogens in patients who received a full Rotarix vaccination regimen (2 doses) and were diagnosed with acute gastroenteritis. These patients were designated as having suspected rotavirus vaccine breakthrough cases (referred to here as breakthrough cases), and their samples were analyzed using both NGS and RT-qPCR. Accordingly, we selected 102 samples collected from 2007 to 2018 (see Materials and Methods).

**FIG 1:**
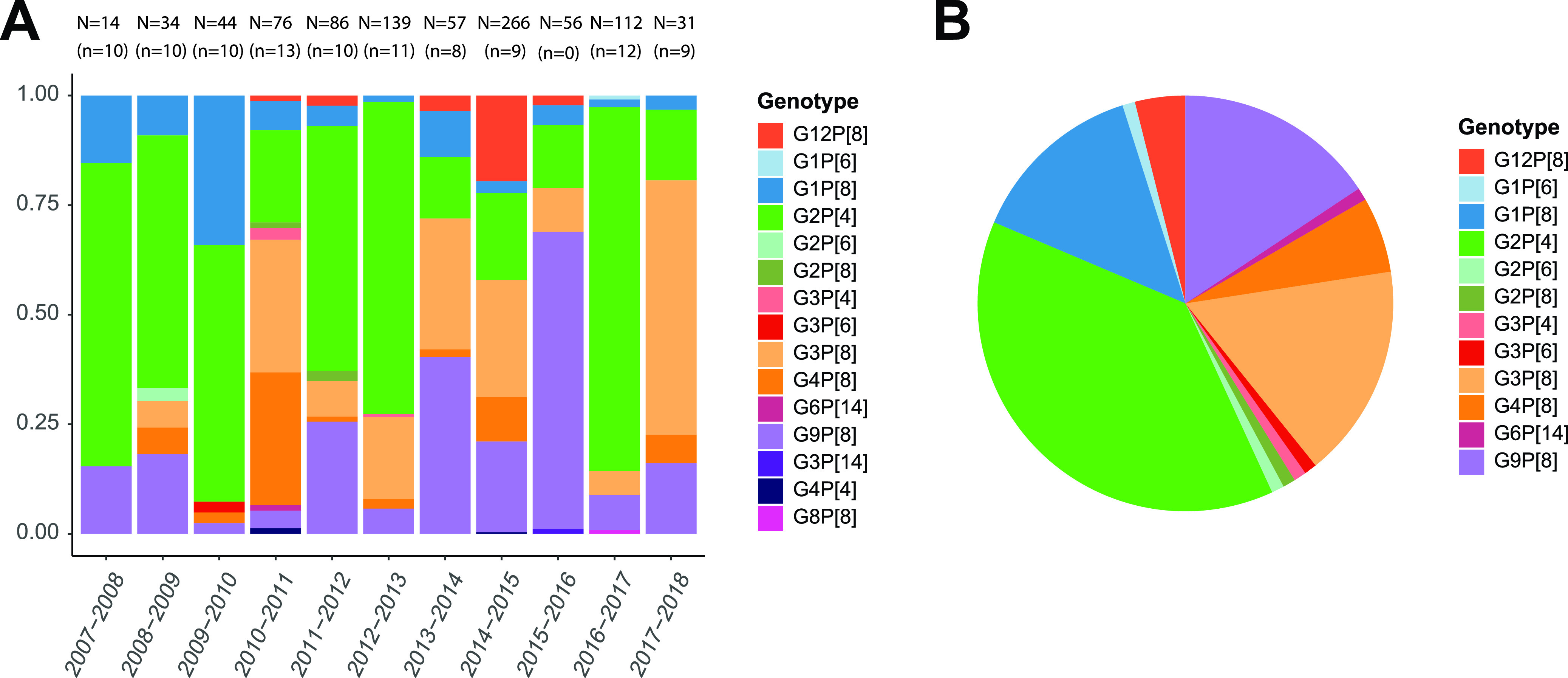
G/P-genotype distribution of the rotavirus vaccine breakthrough strains collected between 2007 and 2018 (postvaccination period). (A) Relative proportions of the rotavirus genotypes per season for all known vaccine breakthrough cases (*n* = 915), with the absolute numbers of cases (N) and the selected number of cases (n) indicated above each bar. (B) Genotype distribution of the 102 samples selected for further analyses.

Figure 1A shows the G/P genotype distributions of all known breakthrough cases, and Fig. 1B shows the genotypes of the 102 selected samples, reflecting the overall breakthrough G and P genotype distribution. Among the 15 detected genotype combinations, the G2P[4], G9P[8], G3P[8], G1P[8], G4P[8], and G12P[8] genotypes were the most common.

### NGS-based identification of coinfecting eukaryotic viral families.

**FIG 2:**
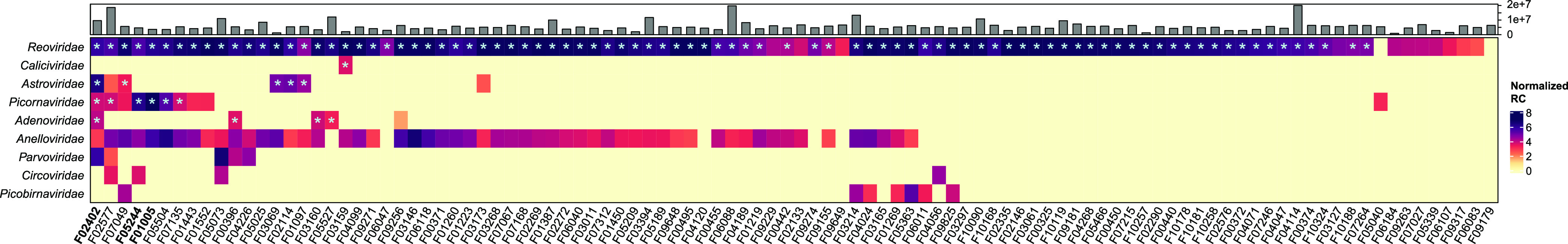
Abundance heat map of the identified human eukaryotic virus families in 102 samples by NGS. The log_10_-transformed and length-normalized NGS read counts (RC) are shown. The total numbers of quality-controlled reads per sample are shown on top. An asterisk indicates that a (nearly) complete genome was obtained from the virus.

To obtain an unbiased view of the viruses in these 102 samples, we purified virus-like particles and subjected them to Illumina sequencing. The relative abundances of identified eukaryotic viral families are shown in Fig. 2. Rotavirus was prevalent and abundant in the majority of the samples. In 18.6% of the samples, we detected nonrotavirus (alternative) eukaryotic virus families containing members known to cause gastroenteritis in humans, namely, *Picornaviridae* (10%), *Astroviridae* (7%), *Adenoviridae* (5%), and *Caliciviridae* (1%). Furthermore, we also detected members of *Anelloviridae* (54%), *Picobirnaviridae* (7%), *Parvoviridae* (5%), and *Circoviridae* (4%); however, they were not included in the downstream analyses, as their association with gastroenteritis is either weak or nonexistent. Some samples contained multiple enteric viruses, and in some cases, the viral read abundance was higher for alternative viral pathogens than for rotavirus (Fig. 2, boldface).

### No unusual rotavirus genotypes or genotype constellations.

**FIG 3:**
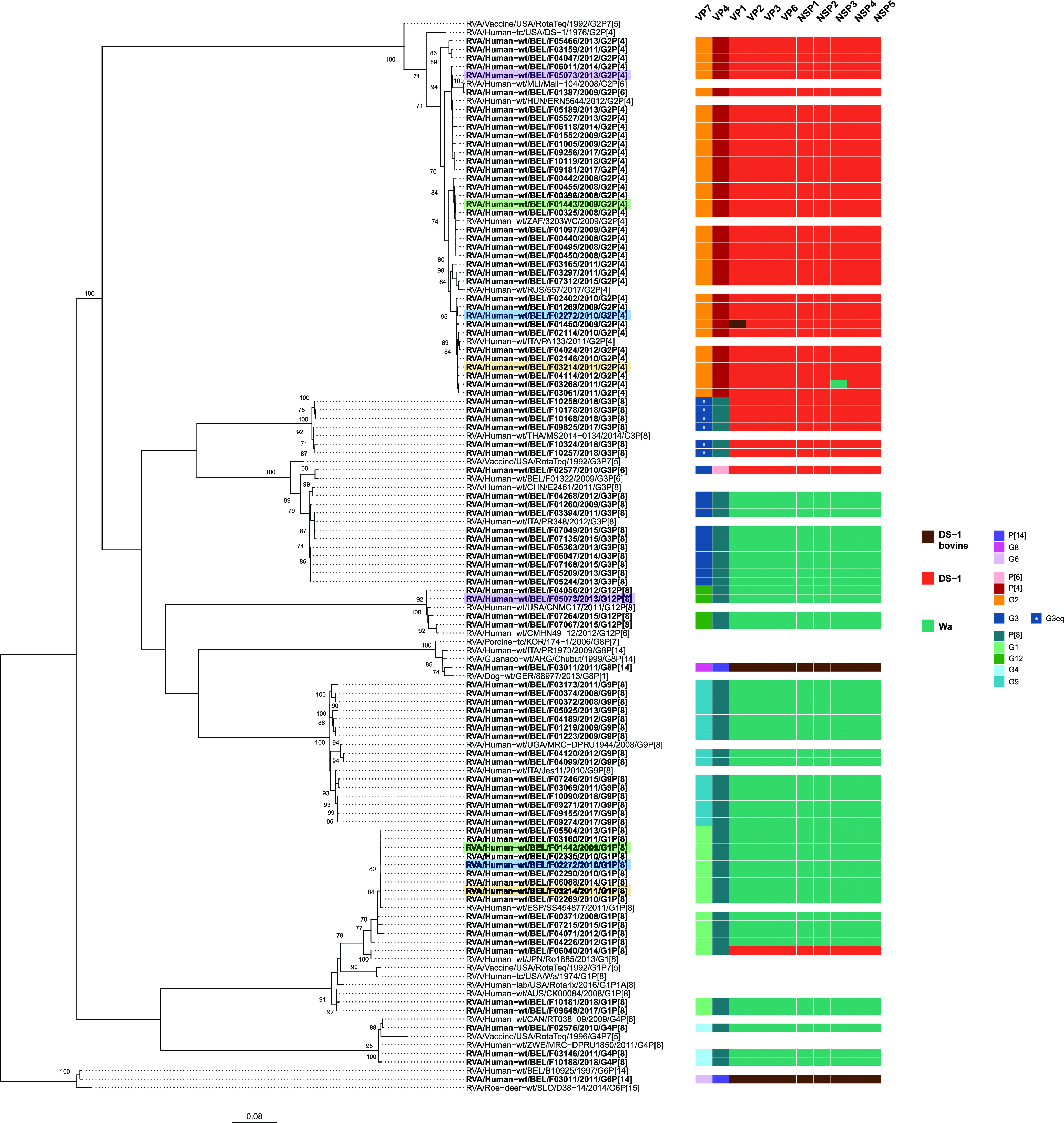
Maximum-likelihood tree of the VP7 gene of the detected rotavirus sequences (in boldface) with reference rotavirus genomes. The genotype constellations of the detected strains are shown on the right. Samples harboring multiple genotype constellations (rotavirus coinfections) are highlighted. Only the bootstrap values above 70% are shown. The branch lengths are drawn to scale and represent the nucleotide substitutions per site.

Next, we evaluated the genotypes and phylogenetic relationships of rotavirus gene segments. The full genotype constellations of 93 (out of 102) human rotaviruses could be determined (Fig. 2, asterisks). Like most of the circulating human rotavirus strains, they possessed either the Wa (51%) or DS-1-like (48%) genotype constellation, except for a single bovine-like genotype constellation in combination with the G6/G8 and P[14] genotypes (Fig. 3). The G1 (*n* = 16), G3 (*n* = 11), G4 (*n* = 4), G9 (*n* = 14), and G12 (*n* = 4) genotypes were mostly found in combination with P[8] and a Wa-like genetic backbone, whereas G2 (*n* = 37) and G3eq (equine-like G3 lineage) (*n* = 6) were linked with a DS-1 backbone. We also observed 3 intergenogroup reassortant rotaviruses: (i) G1P[8] with a DS-1-like backbone (sample F06040), (ii) Wa-like G3 coupled with P[6] and a DS-1-like backbone (F02577), and (iii) Wa-like T1 NSP3 with G2P[4] and a DS-1-like backbone (F03268). We further identified coinfections with Wa-like and DS-1-like strains in 4 samples (Fig. 3, highlighted strain names). In addition, we did not detect vaccine-derived reads in any of the samples (Rotarix and RotaTeq).

The phylogenetic tree of VP7 revealed close genetic relatedness to typical human rotaviruses, with high nucleotide identities (NI) (95 to 99%) (Fig. 3). The VP7 of the double-reassortant DS-1-like G1P[8] (F06040) was closely related to a Japanese double-reassortant strain (42). The DS-1-like G3P[6] (F02577) showed close genetic similarity to another G3P[6] Belgian strain detected in 2009 (43). The 6 G3 strains with a DS-1-like genotype constellation clustered with equine-like G3 strains. There was one sample (F03011) possessing both the G6 and G8 genotypes, clustering closely with other animal or animal-like human strains.

As mentioned above, the VP4 gene and the remaining 9 segments had either a Wa-like or DS-1-like genotype constellation and mostly presented a low genetic diversity compared to currently circulating rotaviruses (Fig. S1 to S10). The 9 segments of the animal-derived F03011 were closely related to bovine-like rotavirus genotypes, as well as zoonotic human strains (44). Interestingly, the VP1 gene of a DS-1-like G2P[4] strain (F01450) clustered with a zoonotic human strain, within the same monophyletic group as the F03011 strain (Fig. S2). Overall, genotype constellations and phylogenetic analysis revealed that the breakthrough strains shared close genetic relatedness to the commonly circulating human rotaviruses in Belgium and worldwide.

### Several variants of common viral enteropathogens.

**FIG 4:**
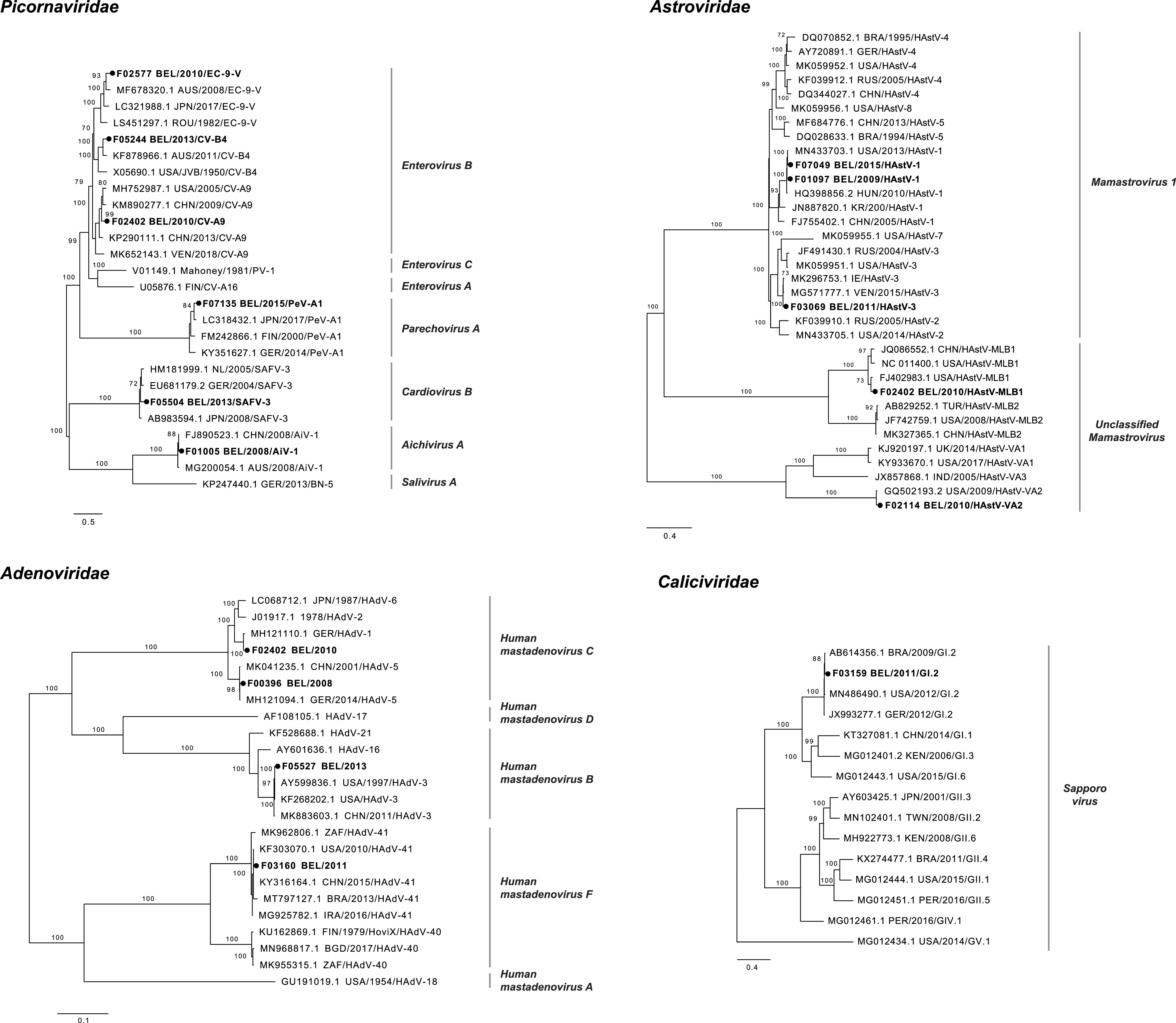
Maximum likelihood trees of the complete genomes of picornaviruses, astroviruses, adenoviruses, and sapoviruses. The strains identified in this study are indicated with boldface type and dark circles. The species are shown next to the branches. Only bootstrap values above 70% are shown. The branch lengths are drawn to scale and represent the number of nucleotide substitutions per site.

For several alternative enteric viruses, (nearly) complete genomes could be obtained for further evolutionary analyses (Fig. 2, asterisks). Nine types within *Picornaviridae* were detected, namely, coxsackievirus A9 (CV-A9), coxsackievirus A4 (CV-A4), coxsackievirus B2 (CV-B2), coxsackievirus B4 (CV-B4), echovirus E9 (EC-9-V), human parechovirus 1 (PeV-A1), Saffold virus 3 (SAFV-3), Aichivirus 1 (AiV-1), and human rhinovirus 78 (RV-A78). The phylogenetic analysis showed that the AiV-1 and PeV-A1 strains clustered closely with known Southeast Asian strains with high NI (99% and 94%, respectively) (Fig. 4) (45). However, several other picornaviruses showed a higher genetic diversity; e.g., enteroviruses CV-A9, CV-B4, and EC-9-V and cardiovirus SAFV-3 shared 87 to 91% NI with their closest references. The genomes assigned to *Astroviridae* were closely related to either the classic human astrovirus strains (HAstV-1 and HAstV-3; 98 to 99% NI) or recently described divergent astroviruses (MLB1 and VA2; 98 to 99% NI) (Fig. 4). Among the *Adenoviridae*, only a single enteric human adenovirus (human mastadenovirus F, HAdV-41) was identified, which clustered with strains detected worldwide with high genetic relatedness (99%) (Fig. 4). Three nonenteric human adenoviruses, classified as human mastadenovirus C (HAdV-C) and human mastadenovirus B (HAdV-B), were also identified. HAdV-C strains clustered closely to either European HAdV-1 or HAdV-5 strains (99% NI). Another strain formed a clade with North American and Southeast Asian HAdV-3 strains with high NI (99%).

Even though no noroviruses were detected by NGS; a single sapovirus (SV) GI.2, which is another member of the *Caliciviridae*, formed a clade with the strains from around the world (98 to 99% NI) (Fig. 4).

### Fifteen enteropathogens identified with RT-qPCR.

**FIG 5:**
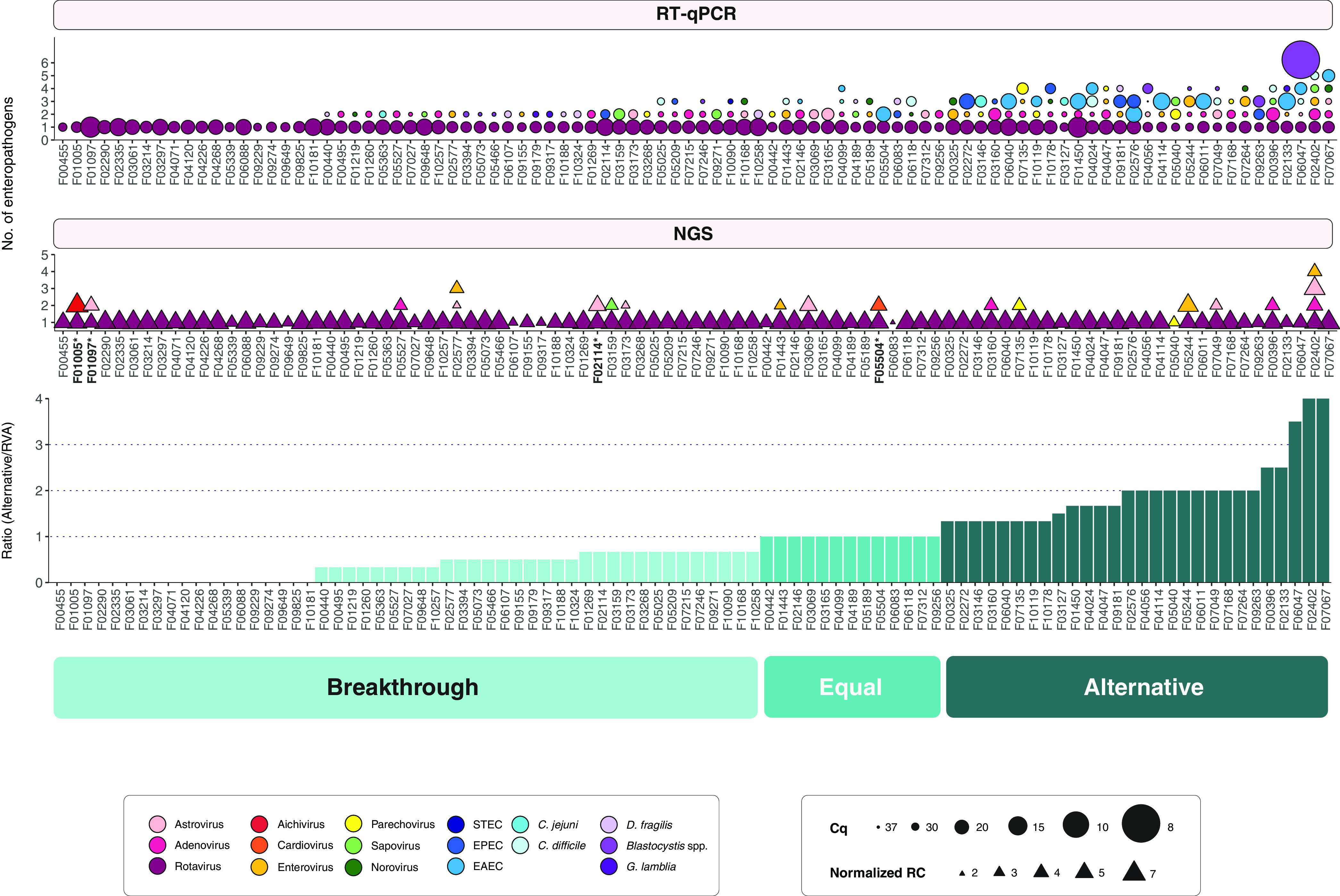
Relative number of the enteropathogens detected in rotavirus breakthrough cases (top) and classification into gastroenteritis etiology groups (bottom). The top panel shows the *C_q_* values based on the RT-qPCR results. The normalized NGS read counts (RC) per pathogen are shown in the middle panel. Different types of detected pathogens are shown in different colors: pink to green, viruses; blue, bacteria; purple, parasites. In the bottom, the grouping of the potential etiology of the gastroenteritis is shown, which is based on the given scores (see Materials and Methods). Samples marked with an asterisk contain a viral pathogen(s) which was not detected by RT-qPCR.

To screen for nonviral enteropathogens (e.g., bacteria and parasites) and to achieve a more sensitive detection for viruses, RT-qPCR was performed on 92 samples for which sufficient material was available. Consequently, 15 out of the 26 (58%) pathogens tested for were detected: rotavirus, adenovirus, astrovirus, picornavirus (enterovirus and parechovirus), sapovirus, norovirus, EPEC (atypical and typical), EAEC, STEC, C. jejuni, C. difficile, *Blastocystis* spp., *D. fragilis*, and G. lamblia (Fig. 5). Conversely, HEV, Salmonella, Y. enterocolitica, Y. pseudotuberculosis, ETEC, EIEC, *Cryptosporidium*, E. histolytica, S. stercoralis, *Schistosoma*, and *Microsporidium* were not detected. RT-qPCR results and demographics of enteropathogen-positive patients are shown in Tables S2 and S3. Rotavirus was detected in all samples, and adenovirus (32%) and enterovirus (15%) were the most common coinfecting viruses (Table S4A). Pathogenic bacteria were identified in 35 samples, whereas protozoan parasites were found in 21 samples. Moreover, there were in total 31 combinations of coinfection consisting of virus-virus, virus-bacterium, virus-parasite, or virus-bacterium-parasite, and the first two accounted for the majority of the coinfections (Table S4B). The most common co-occurring enteropathogens were adenovirus and EAEC (4%), followed by norovirus and enterovirus (2%), and adenovirus and EPEC (2%). Overall, 36% of the samples harbored 1 coinfecting enteropathogen, 27% had 2 enteropathogens, 13% had 3 enteropathogens, and 3% had 4 enteropathogens alongside rotavirus. In the end, combined data from NGS and RT-qPCR showed that there was at least one coinfecting pathogen in 80% of the rotavirus-positive samples.

### Rotavirus is most likely not the cause of gastroenteritis in almost a third of the cases.

After determining the prevalence of enteropathogens in breakthrough samples, we visualized the relative pathogen quantifications per sample, using the *C_q_* values (RT-qPCR) and the relative abundances (NGS) (Fig. 5, top). Relying on the RT-qPCR results, we attempted to assess the role of rotavirus in gastroenteritis etiology and developed a scoring scheme. Accordingly, each pathogen-specific RT-qPCR assay was given a score (see Materials and Methods; Table S6), and the ratio of the alternative-pathogen scores over the rotavirus score was calculated for each patient. Based on this ratio, we designated each sample as representing a true breakthrough case (breakthrough) (ratio < 1), equal contribution of rotavirus and alternative enteropathogen(s) (equal) (ratio = 1), or alternative etiology for gastroenteritis (alternative) (ratio > 1) (Fig. 5, bottom). It is important to note that, as this classification took only RT-qPCR data into account, it excluded viruses that were not included in the RT-qPCR panel, yet were detected by NGS (Fig. 5, samples with asterisks). Among the 3 samples that possessed such strains, a lower relative abundance of astroviruses (F01097 and F02114) and cardiovirus (F05504) was found in comparison to rotavirus (Fig. 2), validating their representation in the breakthrough group. On the other hand, there was an ∼8-fold-higher relative abundance of Aichivirus than rotavirus in F01005, which would place this patient in the alternative gastroenteritis etiology group. This evaluation finally resulted in 50 patients (54.3%) with a true vaccine breakthrough infection, 13 patients (14.1%) with equal contribution of rotavirus and alternative pathogen(s) to gastroenteritis, and 29 patients (31.5%) having an alternative etiology. Moreover, among the patients that possibly had an alternative gastroenteritis cause, 15 of them harbored more than 3 coinfecting agents (Fig. 5; Table S4B).

### Discrepancies between detection methods.

Overall, the results of the NGS and RT-qPCR were in accordance (Table S5). RT-qPCR identified 61 additional viral pathogens in comparison to NGS due to higher sensitivity. On the other hand, there were 4 viruses that were detected only by NGS, as they were not included in the RT-qPCR panel (AiV-1, SAFV-3, MLB1, and VA2). For a single sample (F01097), NGS identified HAstV-1, which should also have been identified by the RT-qPCR panel. The RT-qPCR remained negative after retesting of this sample. Further comparison between the HAstV-1 genome and the primers and probes did not show any mismatches which could have explained this discrepancy (data not shown).

## DISCUSSION

### Multiple coinfections in breakthrough cases.

Even though many enteric pathogens have been associated with pediatric gastroenteritis, only a few are routinely screened, hiding the bigger picture. As several of these infections may not be the (main) cause of gastroenteritis, it can be difficult to determine the true causative agent. Furthermore, not much is known about host genetic and immune responses to gastroenteritis disease progression during coinfections. As a first step, it is important to identify all the enteropathogens, which might contribute to the transition of an asymptomatic infection to a symptomatic one or increase the severity of the disease (46). In this study, we estimated the impact of coinfecting enteric pathogens in suspected rotavirus breakthrough infections in Belgian infants. Surprisingly, approximately half of these cases most likely either had an alternative gastroenteritis etiology (31.5%) or were the result of coinfections including rotavirus (14.1%) (Fig. 5). Most coinfections were unique combinations of viruses, bacteria, and parasites, which shows the complexity involved in reaching a correct diagnosis.

Infants are frequently exposed to enteropathogens in households with multiple children and in daycare centers, but also through poor hygiene and contaminated food (47). Multiple coinfections can cause severe and chronic diarrhea in immunocompromised patients, in some cases with fatal outcomes (48). However, since early exposures to microorganisms is also known to be important for a proper training of the infant immune system, the long- and short-term impact of enteric (co)infections should be more thoroughly investigated (49, 50).

### RT-qPCR detection of bacteria and parasites.

Apart from the bacteria typically associated with pediatric gastroenteritis (diarrheagenic E. coli, Campylobacter, toxinogenic C. difficile), there are also atypical pathogens associated with travelling (e.g., EAEC and ETEC) (15). Traveler’s diarrhea-associated EAEC was frequently detected in this study, often in coinfection with other enteropathogens (Table S4). Unfortunately, we did not have access to the immigration or travel history of the patients to assess the import or endemicity of EAEC in Belgium. Other causative agents of gastroenteritis detected here were parasitic protozoa, which are responsible for less than 10% of the infantile gastroenteritis globally (51). The clinical significance of G. lamblia, *Blastocystis* spp., and *D. fragilis* is still disputed, but association with gastrointestinal symptoms and even chronic diarrhea has been reported, especially in immunocompromised patients (52, 53). *Blastocystis* spp. had a very low *C_q_* value in one patient in this study, which might indicate an acute infection (Fig. 5; Table S2).

### NGS detection of viruses.

Viruses are the most common cause of pediatric gastroenteritis and, as expected, they were also the most detected coinfecting pathogens in this study. The NGS analyses allowed us to further classify and characterize the enteric viruses in the suspected breakthrough cases. Consequently, several known (e.g., HAstV-1 and HAdV-41) and divergent (e.g., CV-B4 and CV-A9) enteric viruses were detected in the alternative gastroenteritis group, which are expected and unexpected candidates in infantile diarrhea, respectively (14, 54, 55). Consistent with previous reports, we also detected several picornaviruses at low frequency and with multiple coinfections, such as PeV-A1, AiV-1, and SAFV-3 (56–58) (Table S4A). Another eukaryotic virus family with a strong link to gastroenteritis is *Astroviridae*, and nonclassic astroviruses have been sporadically detected in diarrheic infants with unclear pathogenicity since 2008 (14). We reported here the first Belgian MLB1 strain detected in 2010 in a 7-month-old infant (Fig. 4). It has been suggested that the astrovirus infection rate is decreasing due to the replacement of classic human astroviruses with the novel strains, which are not part of the screening (14). Even though enteric adenoviruses (type 40/41) are one of the leading causes of gastroenteritis in developed settings (55), we identified nonenteric adenoviruses more frequently (e.g., HAdV-B and HAdV-C) (Fig. 4). Similar observations were made previously in diarrheic cases in Southeast Asia (55), even though subgroup B and C adenoviruses are commonly associated with respiratory infections (59). Few members of the *Caliciviridae* (including the globally circulating SV GI.2) were detected at low prevalence in our study, which was rather unexpected, as they are frequently reported in gastroenteritis (15, 60).

### Comparison of NGS and RT-qPCR detection methods.

As mentioned above, we opted for a combined RT-qPCR and NGS method in this study, and the two approaches yielded similar results. RT-qPCR, as expected, was more sensitive, whereas NGS identified divergent viruses not covered in the RT-qPCR panel and allowed further molecular strain characterization. One of the additional detections by RT-qPCR was noroviruses. Norovirus is reportedly displacing rotavirus as the most common cause of diarrhea hospitalization in settings where rotavirus vaccines are highly effective, such as Belgium (61–63). Identified noroviruses mostly belonged to the GII genogroup, which aligns with the reports on the expansion of the GII.4 since the mid-2000s (64). Furthermore, detection of a classic astrovirus (HAstV-1) by NGS but not by RT-qPCR was an unanticipated result, which might be caused by the additional freeze-thaw step between implementation of NGS and RT-qPCR methods, leading to RNA degradation. Overall, we showed that NGS and RT-qPCR are complementary approaches for the detection and genomic characterization of genetically diverse viral genomes. Moreover, even though the NGS data do not prove that the detected viruses are actually enteropathogens, including nonclassic astrovirus surveillance to investigate their spread and clinical significance should be considered.

### NGS characterization of suspected rotavirus breakthrough strains.

Since rotavirus genotype diversity changes temporally, and its segmented genome makes it prone to reassortments, unusual genotype constellations can emerge and escape vaccine-induced immunity (65). Especially following the mass introduction of rotavirus vaccines, selection of vaccine escape mutants has been a concerning issue (66–68). Our investigation of rotavirus genotypes in the suspected breakthrough infections revealed a pattern similar to that in previous observations, with common detection of Wa and DS-1-like genotype constellations and a lack of evidence of possible vaccine escape mutants (6).

Due to the likely fitness cost to change an established genotype constellation, it was not surprising that only 3 reassortant strains were detected (69) (Fig. 3). Double-reassortant DS-1-like G1P[8] rotaviruses have emerged in Southeast Asia, but they have spread globally in the postvaccine period (70–72). In this study, we report a double reassortant G1P[8] in Belgium that was closely related to a Japanese reassortant G1P[8] (70). The genotype constellation of the Belgian G1P[8] and several G3P[8] strains were also related to equine-like G3P[8] strains. The equine-like strains have already been reported worldwide and they are emerging in Europe (73–77). Another reassortant was a G3P[6] with a DS-1-like backbone, a genotype combination previously described in Belgium in 2009 (43). The G3P[6] type is detected in low frequencies but should be monitored, as it is also fully heterotypic relative to the Rotarix vaccine.

Another driver of genetic diversity in rotavirus population is zoonotic transmissions. Here, we describe a G6P[14] strain (from patient F03011) that was almost identical to the Belgian B10925 strain obtained from a diarrheic infant in 1997 (78) (Fig. 3; Fig. S2 to S10). It was speculated that livestock species are the most likely origin of this infection. Interestingly, patient F03011 seemed to have a coinfection and also possessed a typical bovine G8 genotype, which was phylogenetically closely related to a zoonotic human strain from Italy (44). Overall, rotavirus analysis yielded a low frequency of intergenogroup reassortments and zoonotic transmissions and no unusual genotypes in suspected breakthrough strains in comparison to the circulating human rotaviruses.

### Limitations.

There were several limitations to this study. We did not consider that gastroenteritis can also be noninfectious. Sample selection was nonrandom, and we did not have a control group without acute gastroenteritis in order to associate clinical significance with frequent enteropathogen detection. We also did not have complete information on the epidemiological variables, which could be tested for associations to gastroenteritis etiology groups. Moreover, several identified pathogens are regularly detected in (healthy) infants, and diagnostic testing might not always be relevant. For example, C. difficile is considered a part of the commensal microbiota in infants younger than 2 years of age (79), and the oldest patient that was C. difficile positive here was 16 months old. In addition, RT-qPCR can also detect virus shedding during asymptomatic infections, and even though there are several studies attempting to establish a *C_q_* value cutoff for detection of a symptomatic infection, it is difficult to link viral load with disease (80, 81). On the other hand, several studies have reported a positive correlation between viral load and disease severity, supporting the inference of clinical outcome using *C_q_* values in this paper (82, 83). Despite various limitations, in this proof-of-concept study, we demonstrated multiple coinfections in gastroenteritis cases which were initially classified as rotavirus vaccine breakthrough infections.

## References

[1] Burnett E, Parashar UD, Tate JE. 2020. Global impact of rotavirus vaccination on diarrhea hospitalizations and deaths among children <5 years old: 2006–2019. J Infect Dis 222:1731–1739. 10.1093/infdis/jiaa081. 32095831 PMC7483971

[2] Estes MK, Palmer EL, Obijeski JF. 1983. Rotaviruses: a review. Curr Top Microbiol Immunol 105:1917–1974. 10.1007/978-3-642-69159-1_3 6313296

[3] Matthijnssens J, Ciarlet M, Rahman M, Attoui H, Bányai K, Estes MK, Gentsch JR, Iturriza-Gómara M, Kirkwood CD, Martella V, Mertens PPC, Nakagomi O, Patton JT, Ruggeri FM, Saif LJ, Santos N, Steyer A, Taniguchi K, Desselberger U, Van Ranst M. 2008. Recommendations for the classification of group A rotaviruses using all 11 genomic RNA segments. Arch Virol 153:1621–1629. 10.1007/s00705-008-0155-1. 18604469 PMC2556306

[4] Matthijnssens J, Ciarlet M, Heiman E, Arijs I, Delbeke T, McDonald SM, Palombo EA, Iturriza-Gómara M, Maes P, Patton JT, Rahman M, Ranst MV. 2008. Full genome-based classification of rotaviruses reveals a common origin between human Wa-like and porcine rotavirus strains and human DS-1-like and bovine rotavirus strains. J Virol 82:3204–3219. 10.1128/JVI.02257-07. 18216098 PMC2268446

[5] Burnett E, Parashar UD, Tate JE. 2020. Real-world effectiveness of rotavirus vaccines, 2006–19: a literature review and meta-analysis. Lancet Glob Health 8:e1195–e1202. 10.1016/S2214-109X(20)30262-X. 32827481 PMC8097518

[6] Zeller M, Rahman M, Heylen E, De Coster S, De Vos S, Arijs I, Novo L, Verstappen N, Van Ranst M, Matthijnssens J. 2010. Rotavirus incidence and genotype distribution before and after national rotavirus vaccine introduction in Belgium. Vaccine 28:7507–7513. 10.1016/j.vaccine.2010.09.004. 20851085

[7] Gurgel RQ, Cuevas LE, Vieira SCF, Barros VCF, Fontes PB, Salustino EF, Nakagomi O, Nakagomi T, Dove W, Cunliffe N, Hart CA. 2007. Predominance of rotavirus P[4]G2 in a vaccinated population, Brazil. Emerg Infect Dis 13:1571–1573. 10.3201/eid1310.070412. 18258011 PMC2851506

[8] Thanh HD, Tran VT, Lim I, Kim W. 2018. Emergence of human G2P[4] rotaviruses in the post-vaccination era in South Korea: footprints of multiple interspecies re-assortment events. Sci Rep 8:6011. 10.1038/s41598-018-24511-y. 29662148 PMC5902508

[9] Roczo-Farkas S, Kirkwood CD, Cowley D, Barnes GL, Bishop RF, Bogdanovic-Sakran N, Boniface K, Donato CM, Bines JE. 2018. The impact of rotavirus vaccines on genotype diversity: a comprehensive analysis of 2 decades of Australian surveillance data. J Infect Dis 218:546–554. 10.1093/infdis/jiy197. 29790933

[10] Bibera GL, Chen J, Pereira P, Benninghoff B. 2020. Dynamics of G2P[4] strain evolution and rotavirus vaccination: a review of evidence for Rotarix. Vaccine 38:5591–5600. 10.1016/j.vaccine.2020.06.059. 32651115

[11] Bartlett AV, Reves RR, Pickering LK. 1988. Rotavirus in infant-toddler day care centers: epidemiology relevant to disease control strategies. J Pediatr 113:435–441. 10.1016/s0022-3476(88)80624-3. 2842485 PMC7130773

[12] Hoa Tran TN, Trainor E, Nakagomi T, Cunliffe NA, Nakagomi O. 2013. Molecular epidemiology of noroviruses associated with acute sporadic gastroenteritis in children: global distribution of genogroups, genotypes and GII.4 variants. J Clin Virol 56:185–277. 10.1016/j.jcv.2012.11.011. 23218993

[13] Uhnoo I, Wadell G, Svensson L, Johansson ME. 1984. Importance of enteric adenoviruses 40 and 41 in acute gastroenteritis in infants and young children. J Clin Microbiol 20:365–372. 10.1128/jcm.20.3.365-372.1984. 6092424 PMC271331

[14] Bosch A, Pintó RM, Guix S. 2014. Human astroviruses. Clin Microbiol Rev 27:1048–1074. 10.1128/CMR.00013-14. 25278582 PMC4187635

[15] Elliott EJ. 2007. Acute gastroenteritis in children. BMJ 334:35–40. 10.1136/bmj.39036.406169.80. 17204802 PMC1764079

[16] Matthijnssens J, Zeller M, Heylen E, Coster SD, Vercauteren J, Braeckman T, Herck KV, Meyer N, PirÇon J-Y, Soriano-Gabarro M, Azou M, Capiau H, Koster JD, Maernoudt A-S, Raes M, Verdonck L, Verghote M, Vergison A, Damme PV, Ranst MV. 2014. Higher proportion of G2P[4] rotaviruses in vaccinated hospitalized cases compared with unvaccinated hospitalized cases, despite high vaccine effectiveness against heterotypic G2P[4] rotaviruses. Clin Microbiol Infect 20:O702–O710. 10.1111/1469-0691.12612. 24580887

[17] Praharaj I, Platts-Mills JA, Taneja S, Antony K, Yuhas K, Flores J, Cho I, Bhandari N, Revathy R, Bavdekar A, Rongsen-Chandola T, McMurry T, Houpt ER, Kang G. 2019. Diarrheal etiology and impact of coinfections on rotavirus vaccine efficacy estimates in a clinical trial of a monovalent human–bovine (116E) oral rotavirus vaccine, Rotavac, India. Clin Infect Dis 69:243–250. 10.1093/cid/ciy896. 30335135 PMC6603264

[18] Mokomane M, Tate JE, Steenhoff AP, Esona MD, Bowen MD, Lechiile K, Pernica JM, Kasvosve I, Parashar UD, Goldfarb DM. 2018. Evaluation of the influence of gastrointestinal co-infections on rotavirus vaccine effectiveness in Botswana. Pediatr Infect Dis J 37:e58–e62. 10.1097/INF.0000000000001828. 29189612 PMC5807168

[19] Zhang S-X, Zhou Y-M, Xu W, Tian L-G, Chen J-X, Chen S-H, Dang Z-S, Gu W-P, Yin J-W, Serrano E, Zhou X-N. 2016. Impact of co-infections with enteric pathogens on children suffering from acute diarrhea in southwest China. Infect Dis Poverty 5:64. 10.1186/s40249-016-0157-2. 27349521 PMC4922062

[20] Conceição-Neto N, Zeller M, Lefrère H, De Bruyn P, Beller L, Deboutte W, Yinda CK, Lavigne R, Maes P, Ranst MV, Heylen E, Matthijnssens J. 2015. Modular approach to customise sample preparation procedures for viral metagenomics: a reproducible protocol for virome analysis. Sci Rep 5:16532. 10.1038/srep16532. 26559140 PMC4642273

[21] Yinda CK, Zeller M, Conceição-Neto N, Maes P, Deboutte W, Beller L, Heylen E, Ghogomu SM, Van Ranst M, Matthijnssens J. 2016. Novel highly divergent reassortant bat rotaviruses in Cameroon, without evidence of zoonosis. Sci Rep 6:34209. 10.1038/srep34209. 27666390 PMC5035928

[22] Bolger AM, Lohse M, Usadel B. 2014. Trimmomatic: a flexible trimmer for Illumina sequence data. Bioinformatics 30:2114–2120. 10.1093/bioinformatics/btu170. 24695404 PMC4103590

[23] Nurk S, Meleshko D, Korobeynikov A, Pevzner PA. 2017. metaSPAdes: a new versatile metagenomic assembler. Genome Res 27:824–834. 10.1101/gr.213959.116. 28298430 PMC5411777

[24] Bolduc B, Roux S. 2017. Clustering viral genomes in iVirus. protocols.io. 10.17504/protocols.io.gwebxbe.

[25] Buchfink B, Xie C, Huson DH. 2015. Fast and sensitive protein alignment using DIAMOND. Nat Methods 12:59–60. 10.1038/nmeth.3176. 25402007

[26] Li H, Durbin R. 2009. Fast and accurate short read alignment with Burrows–Wheeler transform. Bioinformatics 25:1754–1760. 10.1093/bioinformatics/btp324. 19451168 PMC2705234

[27] Li H, Handsaker B, Wysoker A, Fennell T, Ruan J, Homer N, Marth G, Abecasis G, Durbin R, 1000 Genome Project Data Processing Subgroup. 2009. The Sequence Alignment/Map format and SAMtools. Bioinformatics 25:2078–2079. 10.1093/bioinformatics/btp352. 19505943 PMC2723002

[28] Wickham H. 2007. Reshaping data with the reshape package. J Stat Soft 21:1–20. 10.18637/jss.v021.i12.

[29] Wickham H, Averick M, Bryan J, Chang W, McGowan L, François R, Grolemund G, Hayes A, Henry L, Hester J, Kuhn M, Pedersen T, Miller E, Bache S, Müller K, Ooms J, Robinson D, Seidel D, Spinu V, Takahashi K, Vaughan D, Wilke C, Woo K, Yutani H. 2019. Welcome to the Tidyverse. J Open Source Softw 4:1686. 10.21105/joss.01686.

[30] Gu Z, Eils R, Schlesner M. 2016. Complex heatmaps reveal patterns and correlations in multidimensional genomic data. Bioinformatics 32:2847–2849. 10.1093/bioinformatics/btw313. 27207943

[31] Kroneman A, Vennema H, Deforche K, Aoort HVD, Peñaranda S, Oberste MS, Vinjé J, Koopmans M. 2011. An automated genotyping tool for enteroviruses and noroviruses. J Clin Virol 51:121–125. 10.1016/j.jcv.2011.03.006. 21514213

[32] Pickett BE, Sadat EL, Zhang Y, Noronha JM, Squires RB, Hunt V, Liu M, Kumar S, Zaremba S, Gu Z, Zhou L, Larson CN, Dietrich J, Klem EB, Scheuermann RH. 2012. ViPR: an open bioinformatics database and analysis resource for virology research. Nucleic Acids Res 40:D593–D598. 10.1093/nar/gkr859. 22006842 PMC3245011

[33] Wheeler DL, Barrett T, Benson DA, Bryant SH, Canese K, Chetvernin V, Church DM, DiCuccio M, Edgar R, Federhen S, Geer LY, Kapustin Y, Khovayko O, Landsman D, Lipman DJ, Madden TL, Maglott DR, Ostell J, Miller V, Pruitt KD, Schuler GD, Sequeira E, Sherry ST, Sirotkin K, Souvorov A, Starchenko G, Tatusov RL, Tatusova TA, Wagner L, Yaschenko E. 2007. Database resources of the National Center for Biotechnology Information. Nucleic Acids Res 35:D5–D12. 10.1093/nar/gkl1031. 17170002 PMC1781113

[34] Edgar RC. 2004. MUSCLE: multiple sequence alignment with high accuracy and high throughput. Nucleic Acids Res 32:1792–1797. 10.1093/nar/gkh340. 15034147 PMC390337

[35] Kumar S, Stecher G, Tamura K. 2016. MEGA7: molecular evolutionary genetics analysis version 7.0 for bigger datasets. Mol Biol Evol 33:1870–1874. 10.1093/molbev/msw054. 27004904 PMC8210823

[36] Katoh K, Rozewicki J, Yamada KD. 2019. MAFFT online service: multiple sequence alignment, interactive sequence choice and visualization. Brief Bioinform 20:1160–1166. 10.1093/bib/bbx108. 28968734 PMC6781576

[37] Posada D. 2008. jModelTest: phylogenetic model averaging. Mol Biol Evol 25:1253–1256. 10.1093/molbev/msn083. 18397919

[38] Kozlov AM, Darriba D, Flouri T, Morel B, Stamatakis A. 2019. RAxML-NG: a fast, scalable and user-friendly tool for maximum likelihood phylogenetic inference. Bioinformatics 35:4453–4455. 10.1093/bioinformatics/btz305. 31070718 PMC6821337

[39] Yu G, Smith DK, Zhu H, Guan Y, Lam TT-Y. 2017. ggtree: an R package for visualization and annotation of phylogenetic trees with their covariates and other associated data. Methods Ecol Evol 8:28–36. 10.1111/2041-210X.12628.

[40] Osterhaus ADME, Vedder EJ. 1988. Identification of virus causing recent seal deaths. Nature 335:20–20. 10.1038/335020a0. 3412456

[41] Schmittgen TD, Livak KJ. 2008. Analyzing real-time PCR data by the comparative CT method. Nat Protoc 3:1101–1108. 10.1038/nprot.2008.73. 18546601

[42] Ihira M, Kawamura Y, Miura H, Hattori F, Higashimoto Y, Sugata K, Ide T, Komoto S, Taniguchi K, Yoshikawa T. 2020. Molecular characterization of rotaviruses obtained from patients with rotavirus-associated encephalitis/encephalopathy. Microbiol Immunol 64:541–555. 10.1111/1348-0421.12827. 32511783

[43] Heylen E, Zeller M, Ciarlet M, De Coster S, Van Ranst M, Matthijnssens J. 2013. Complete genetic characterization of human G2P[6] and G3P[6] rotavirus strains. Infect Genet Evol 13:27–35. 10.1016/j.meegid.2012.08.019. 22982160

[44] Medici MC, Tummolo F, Bonica MB, Heylen E, Zeller M, Calderaro A, Matthijnssens J. 2015. Genetic diversity in three bovine-like human G8P[14] and G10P[14] rotaviruses suggests independent interspecies transmission events. J Gen Virol 96:1161–1168. 10.1099/vir.0.000055. 25614586

[45] Yang S, Zhang W, Shen Q, Yang Z, Zhu J, Cui L, Hua X. 2009. Aichi virus strains in children with gastroenteritis, China. Emerg Infect Dis 15:1703–1705. 10.3201/eid1510.090522. 19861087 PMC2866408

[46] Bhavnani D, Goldstick JE, Cevallos W, Trueba G, Eisenberg JNS. 2012. Synergistic effects between rotavirus and coinfecting pathogens on diarrheal disease:eEvidence from a community-based study in northwestern Ecuador. Am J Epidemiol 176:387–395. 10.1093/aje/kws220. 22842722 PMC3499114

[47] Makimaa H, Ingle H, Baldridge MT. 2020. Enteric viral co-infections: pathogenesis and perspective. Viruses 12:904. 10.3390/v12080904. PMC7472086 32824880

[48] Daniel-Wayman S, Fahle G, Palmore T, Green KY, Prevots DR. 2018. Norovirus, astrovirus, and sapovirus among immunocompromised patients at a tertiary care research hospital. Diagn Microbiol Infect Dis 92:143–146. 10.1016/j.diagmicrobio.2018.05.017. 29934072 PMC11036324

[49] Milani C, Duranti S, Bottacini F, Casey E, Turroni F, Mahony J, Belzer C, Delgado Palacio S, Arboleya Montes S, Mancabelli L, Lugli GA, Rodriguez JM, Bode L, de Vos W, Gueimonde M, Margolles A, van Sinderen D, Ventura M. 2017. The first microbial colonizers of the human gut: composition, activities, and health implications of the infant gut microbiota. Microbiol Mol Biol Rev 81:e00036-17. 10.1128/MMBR.00036-17. PMC5706746 29118049

[50] Jatzlauk G, Bartel S, Heine H, Schloter M, Krauss-Etschmann S. 2017. Influences of environmental bacteria and their metabolites on allergies, asthma, and host microbiota. Allergy 72:1859–1867. 10.1111/all.13220. 28600901

[51] Aranda-Michel J, Giannella RA. 1999. Acute diarrhea: a practical review. Am J Med 106:670–676. 10.1016/s0002-9343(99)00128-x. 10378626 PMC7124219

[52] Tan KSW. 2008. New insights on classification, identification, and clinical relevance of Blastocystis spp. Clin Microbiol Rev 21:639–665. 10.1128/CMR.00022-08. 18854485 PMC2570156

[53] Röser D, Simonsen J, Nielsen HV, Stensvold CR, Mølbak K. 2013. Dientamoeba fragilis in Denmark: epidemiological experience derived from four years of routine real-time PCR. Eur J Clin Microbiol Infect Dis 32:1303–1310. 10.1007/s10096-013-1880-2. 23609513

[54] Tapparel C, Siegrist F, Petty TJ, Kaiser L. 2013. Picornavirus and enterovirus diversity with associated human diseases. Infect Genet Evol 14:282–293. 10.1016/j.meegid.2012.10.016. 23201849

[55] Kumthip K, Khamrin P, Ushijima H, Maneekarn N. 2019. Enteric and non-enteric adenoviruses associated with acute gastroenteritis in pediatric patients in Thailand, 2011 to 2017. PLoS One 14:e0220263. 10.1371/journal.pone.0220263. 31369615 PMC6675392

[56] Filippis AMB, de Souza Luna LK, Stöcker A, Almeida PS, Ribeiro TCM, Petersen N, Herzog P, Pedroso C, Huppertz HI, Ribeiro HDC, Baumgarte S, Park SS. 2008. Circulation of 3 lineages of a novel saffold cardiovirus in humans. Emerg Infect Dis 14:1398–1405. 10.3201/eid1409.080570. 18760006 PMC2603095

[57] Olijve L, Jennings L, Walls T. 2018. Human parechovirus: an increasingly recognized cause of sepsis-like illness in young infants. Clin Microbiol Rev 31:e00047-17. 10.1128/CMR.00047-17. 29142080 PMC5740974

[58] Oh D-Y, Silva PA, Hauroeder B, Diedrich S, Cardoso DDP, Schreier E. 2006. Molecular characterization of the first Aichi viruses isolated in Europe and in South America. Arch Virol 151:1199–1206. 10.1007/s00705-005-0706-7. 16421634

[59] Ghebremedhin B. 2014. Human adenovirus: viral pathogen with increasing importance. Eur J Microbiol Immunol 4:26–33. 10.1556/EuJMI.4.2014.1.2. PMC3955829 24678403

[60] Tohma K, Kulka M, Coughlan S, Green KY, Parra GI. 2020. Genomic analyses of human sapoviruses detected over a 40-year period reveal disparate patterns of evolution among genotypes and genome regions. Viruses 12:516. 10.3390/v12050516. PMC7290424 32392864

[61] Koo HL, Neill FH, Estes MK, Munoz FM, Cameron A, DuPont HL, Atmar RL. 2013. Noroviruses: the most common pediatric viral enteric pathogen at a large university hospital after introduction of rotavirus vaccination. J Pediatr Infect Dis Soc 2:57–60. 10.1093/jpids/pis070. PMC3656546 23687584

[62] Bucardo F, Reyes Y, Svensson L, Nordgren J. 2014. Predominance of norovirus and sapovirus in Nicaragua after implementation of universal rotavirus vaccination. PLoS One 9:e98201. 10.1371/journal.pone.0098201. 24849288 PMC4029982

[63] Hemming M, Räsänen S, Huhti L, Paloniemi M, Salminen M, Vesikari T. 2013. Major reduction of rotavirus, but not norovirus, gastroenteritis in children seen in hospital after the introduction of RotaTeq vaccine into the National Immunization Programme in Finland. Eur J Pediatr 172:739–746. 10.1007/s00431-013-1945-3. 23361964 PMC7086648

[64] Lopman B, Vennema H, Kohli E, Pothier P, Sanchez A, Negredo A, Buesa J, Schreier E, Gray J, Gallimore C, Bottiger B, Hedlund K-O, Torvén M, von Bonsdorff C-H, Maunula L, Poljsak-Prijatelj M, Zimsek J, Reuter G, Szücs G, Melegh B, Svennson L, van Duijnhoven Y, Koopmans M, Reacher M, Brown D, Iturriza M. 2004. Increase in viral gastroenteritis outbreaks in Europe and epidemic spread of new norovirus variant. Lancet 363:682–688. 10.1016/S0140-6736(04)15641-9. 15001325

[65] Kirkwood CD. 2010. Genetic and antigenic diversity of human rotaviruses: potential impact on vaccination programs. J Infect Dis 202(Suppl):S43–S48. 10.1086/653548. 20684716

[66] Rasebotsa S, Mwangi PN, Mogotsi MT, Sabiu S, Magagula NB, Rakau K, Uwimana J, Mutesa L, Muganga N, Murenzi D, Tuyisenge L, Jaimes J, Esona MD, Bowen MD, Mphahlele MJ, Seheri ML, Mwenda JM, Nyaga MM. 2020. Whole genome and in-silico analyses of G1P[8] rotavirus strains from pre- and post-vaccination periods in Rwanda. Sci Rep 10:13460. 10.1038/s41598-020-69973-1. 32778711 PMC7417577

[67] Matthijnssens J, Nakagomi O, Kirkwood CD, Ciarlet M, Desselberger U, Ranst MV. 2012. Group A rotavirus universal mass vaccination: how and to what extent will selective pressure influence prevalence of rotavirus genotypes? Expert Rev Vaccines 11:1347–1354. 10.1586/erv.12.105. 23249234

[68] Matthijnssens J, Heylen E, Zeller M, Rahman M, Lemey P, Van Ranst M. 2010. Phylodynamic analyses of rotavirus genotypes G9 and G12 underscore their potential for swift global spread. Mol Biol Evol 27:2431–2436. 10.1093/molbev/msq137. 20522727

[69] McDonald SM, Matthijnssens J, McAllen JK, Hine E, Overton L, Wang S, Lemey P, Zeller M, Van Ranst M, Spiro DJ, Patton JT. 2009. Evolutionary dynamics of human rotaviruses: balancing reassortment with preferred genome constellations. PLoS Pathog 5:e1000634. 10.1371/journal.ppat.1000634. 19851457 PMC2760143

[70] Komoto S, Tacharoenmuang R, Guntapong R, Ide T, Tsuji T, Yoshikawa T, Tharmaphornpilas P, Sangkitporn S, Taniguchi K. 2016. Reassortment of human and animal rotavirus gene segments in emerging DS-1-like G1P[8] rotavirus strains. PLoS One 11:e0148416. 10.1371/journal.pone.0148416. 26845439 PMC4742054

[71] Jere KC, Chaguza C, Bar-Zeev N, Lowe J, Peno C, Kumwenda B, Nakagomi O, Tate JE, Parashar UD, Heyderman RS, French N, Cunliffe NA, Iturriza-Gomara M. 2017. Emergence of double- and triple-gene reassortant G1P[8] rotaviruses possessing a DS-1-like backbone after rotavirus vaccine introduction in Malawi. J Virol 92:e01246-17. 10.1128/JVI.01246-17. PMC5774894 29142125

[72] Luchs A, da Costa AC, Cilli A, Komninakis SCV, Carmona RDCC, Morillo SG, Sabino EC, Timenetsky MDCST. 2019. First detection of DS-1-like G1P[8] double-gene reassortant rotavirus strains on the American continent, Brazil, 2013. Sci Rep 9:2210. 10.1038/s41598-019-38703-7. 30778110 PMC6379365

[73] Esposito S, Camilloni B, Bianchini S, Ianiro G, Polinori I, Farinelli E, Monini M, Principi N. 2019. First detection of a reassortant G3P[8] rotavirus A strain in Italy: a case report in an 8-year-old child. Virol J 16:64. 10.1186/s12985-019-1173-1. 31092258 PMC6521491

[74] Cowley D, Donato CM, Roczo-Farkas S, Kirkwood CD. 2016. Emergence of a novel equine-like G3P[8] inter-genogroup reassortant rotavirus strain associated with gastroenteritis in Australian children. J Gen Virol 97:403–410. 10.1099/jgv.0.000352. 26588920

[75] Katz EM, Esona MD, Betrapally NS, De La Cruz De Leon LA, Neira YR, Rey GJ, Bowen MD. 2019. Whole-gene analysis of inter-genogroup reassortant rotaviruses from the Dominican Republic: emergence of equine-like G3 strains and evidence of their reassortment with locally-circulating strains. Virology 534:114–131. 10.1016/j.virol.2019.06.007. 31228725

[76] Perkins C, Mijatovic-Rustempasic S, Ward ML, Cortese MM, Bowen MD. 2017. Genomic characterization of the first equine-like G3P[8] rotavirus strain detected in the United States. Genome Announc 5:e01341-17. 10.1128/genomeA.01341-17. 29167260 PMC5701485

[77] Tacharoenmuang R, Komoto S, Guntapong R, Upachai S, Singchai P, Ide T, Fukuda S, Ruchusatsawast K, Sriwantana B, Tatsumi M, Motomura K, Takeda N, Murata T, Sangkitporn S, Taniguchi K, Yoshikawa T. 2020. High prevalence of equine-like G3P[8] rotavirus in children and adults with acute gastroenteritis in Thailand. J Med Virol 92:174–186. 10.1002/jmv.25591. 31498444

[78] Matthijnssens J, Potgieter CA, Ciarlet M, Parreño V, Martella V, Bányai K, Garaicoechea L, Palombo EA, Novo L, Zeller M, Arista S, Gerna G, Rahman M, Ranst MV. 2009. Are human P[14] rotavirus strains the result of interspecies transmissions from sheep or other ungulates that belong to the mammalian order Artiodactyla? J Virol 83:2917–2929. 10.1128/JVI.02246-08. 19153225 PMC2655590

[79] Kuiper G-A, van Prehn J, Ang W, Kneepkens F, van der Schoor S, de Meij T. 2017. Clostridium difficile infections in young infants: case presentations and literature review. IDCases 10:7–11. 10.1016/j.idcr.2017.07.005. 28791215 PMC5536825

[80] Corcoran MS, van Well GTJ, van Loo IHM. 2014. Diagnosis of viral gastroenteritis in children: interpretation of real-time PCR results and relation to clinical symptoms. Eur J Clin Microbiol Infect Dis 33:1663–1673. 10.1007/s10096-014-2135-6. 24828003

[81] Phillips G, Lopman B, Tam CC, Iturriza-Gomara M, Brown D, Gray J. 2009. Diagnosing rotavirus A associated IID: using ELISA to identify a cut-off for real time RT-PCR. J Clin Virol 44:242–245. 10.1016/j.jcv.2008.12.001. 19179107

[82] Kang G, Iturriza-Gomara M, Wheeler JG, Crystal P, Monica B, Ramani S, Primrose B, Moses PD, Gallimore CI, Brown DW, Gray J. 2004. Quantitation of group A rotavirus by real-time reverse-transcription-polymerase chain reaction: correlation with clinical severity in children in South India. J Med Virol 73:118–122. 10.1002/jmv.20053. 15042658 PMC2459214

[83] Bennett A, Pollock L, Jere KC, Pitzer VE, Lopman B, Bar-Zeev N, Iturriza-Gomara M, Cunliffe NA. 2020. Duration and density of fecal rotavirus shedding in vaccinated Malawian children with rotavirus gastroenteritis. J Infect Dis 222:2035–2040. 10.1093/infdis/jiz612. 31834930 PMC7661767

