# Supplementary material for: High Prevalence of Coinfecting Enteropathogens in Suspected Rotavirus Vaccine Breakthrough Cases
Source: J Clin Microbiol. 2021 Nov 18;59(12):e01236-21. doi: 10.1128/JCM.01236-21 (PMC8601229; doi:10.1128/JCM.01236-21)
Supplement: Supplemental file 1 — Tables S1 to S6 and Fig. S1 to S10. Download jcm.01236-21-s0001.pdf, PDF file, 0.7 MB [file jcm.01236-21-s0001.pdf]

Supplementary Tables and Figures

Table S1. Demographics and sequencing characteristics of the rotavirus vaccine breakthrough samples (n=102). Median age in months, mean number of raw reads, mean number of QC (quality controlled) reads, total number of raw reads and total number of QC reads are shown at the bottom. The samples marked with asterisks were not part of the RT-qPCR analyses. IQR: interquartile range.

Sample ID	Year of sample collection	Sex	Age at sample collection (month)	Time between last Rotarix vaccination and sample collection (month)	Illumina Sequencing platform	Raw reads	QC reads	Rotavirus genotype
**F00325**	2007-2008	F	15	12	NextSeq	5,453,778	4,406,536	G2P[4]
**F00371***	2007-2008	F	19	16	NextSeq	4,386,252	3,418,164	G1P[8]
**F00372***	2007-2008	M	20	15	NextSeq	3,497,962	2,778,664	G9P[8]
**F00374***	2007-2008	M	20	15	NovaSeq	7,839,812	6,335,850	G9P[8]
**F00396**	2007-2008	M	16	13	NextSeq	6,113,596	5,355,736	G2P[4]
**F00440**	2007-2008	F	12	8	NextSeq	6,464,652	4,286,022	G2P[4]
**F00442**	2007-2008	M	12	7	NextSeq	6,118,504	4,627,708	G2P[4]
**F00450***	2007-2008	F	20	15	NextSeq	4,864,644	4,049,438	G2P[4]
**F00455**	2007-2008	M	17	13	NextSeq	3,969,616	3,428,762	G2P[4]
**F00495**	2007-2008	M	10	5	NextSeq	6,128,198	5,217,640	G2P[4]
**F01005**	2008-2009	M	14	11	NextSeq	4,791,888	3,640,842	G2P[4]
**F01097**	2008-2009	F	11	8	NextSeq	7,554,272	5,545,898	G2P[4]
**F01219**	2008-2009	M	12	10	NextSeq	6,335,984	4,038,934	G9P[8]
**F01223***	2008-2009	M	6	2	NextSeq	5,372,482	4,477,742	G9P[8]
**F01260**	2008-2009	F	24	21	NovaSeq	7,200,048	5,784,752	G3P[8]
**F01269**	2008-2009	F	21	17	NextSeq	6,119,802	5,235,520	G2P[4]
**F01387***	2008-2009	Unk.	8	5	NovaSeq	11,694,442	10,047,412	G2P[6]
**F01443**	2008-2009	F	11	8	NextSeq	5,140,790	4,184,690	G1P[8] and G2P[4]
**F01450**	2008-2009	M	21	17	NextSeq	5,819,420	4,647,978	G2P[4]
**F01552***	2008-2009	M	33	27	NextSeq	5,061,388	4,540,214	G2P[4]
**F02114**	2009-2010	M	20	17	NextSeq	7,047,886	5,400,544	G2P[4]
**F02133**	2009-2010	F	28	25	NextSeq	9,058,684	6,708,042	G2P[4]
**F02146**	2009-2010	M	14	10	NextSeq	6,050,646	5,212,260	G2P[4]
**F02269***	2009-2010	F	21	18	NextSeq	4,627,172	3,914,954	G1P[8]
**F02272**	2009-2010	M	20	16	NextSeq	4,756,342	3,748,464	G1P[8] and G2P[4]
**F02290**	2009-2010	F	33	29	NextSeq	6,839,296	5,487,460	G1P[8]
**F02335**	2009-2010	M	24	21	NextSeq	3,331,466	2,854,282	G1P[8]
**F02402**	2009-2010	F	7	4	NovaSeq	11,067,480	9,607,244	G2P[4]
**F02576**	2009-2010	M	50	44	NextSeq	6,705,882	2,765,556	G4P[8]
**F02577**	2009-2010	M	21	16	NovaSeq	22,180,398	18,674,986	G3P[6]
**F03011***	2010-2011	F	10	6	NextSeq	5,080,516	4,382,778	G6P[14]
**F03061**	2010-2011	M	6	2	NextSeq	6,677,286	5,734,160	G2P[4]
**F03069**	2010-2011	M	9	4	NextSeq	6,312,878	1,187,446	G9P[8]
**F03127**	2010-2011	M	16	13	NextSeq	6,591,098	5,522,696	G3P[4]

**F03146**	2010-2011	F	8	5	NextSeq	5,179,074	4,101,312	G4P[8]
**F03159**	2010-2011	F	6	2	NextSeq	5,613,578	2,050,852	G2P[4]
**F03160**	2010-2011	F	14	10	NextSeq	4,325,616	3,405,244	G1P[8]
**F03165**	2010-2011	M	13	8	NextSeq	4,921,070	4,132,782	G2P[4]
**F03173**	2010-2011	M	11	8	NextSeq	6,084,476	5,103,188	G9P[8]
**F03214**	2010-2011	M	14	11	NovaSeq	15,014,930	13,325,050	G1P[8] and G2P[4]
**F03268**	2010-2011	F	16	11	NextSeq	6,134,956	4,941,444	G2P[4]
**F03297**	2010-2011	F	59	55	NextSeq	5,729,534	5,042,012	G2P[4]
**F03394**	2010-2011	F	35	32	NextSeq	14,066,080	11,724,442	G3P[8]
**F04024**	2011-2012	M	7	4	NextSeq	6,816,900	5,754,890	G2P[4]
**F04047**	2011-2012	M	21	18	NextSeq	5,306,444	4,404,828	G2P[4]
**F04056**	2011-2012	F	34	31	NextSeq	7,179,600	5,516,678	G12P[8]
**F04071**	2011-2012	M	28	25	NextSeq	4,663,996	3,569,374	G1P[8]
**F04099**	2011-2012	M	9	5	NextSeq	6,644,622	5,281,288	G9P[8]
**F04114**	2011-2012	M	20	17	NovaSeq	22,482,210	19,971,762	G2P[4]
**F04120**	2011-2012	F	11	5	NextSeq	5,387,180	4,466,628	G9P[8]
**F04189**	2011-2012	M	21	16	NovaSeq	9,725,706	8,275,838	G9P[8]
**F04226**	2011-2012	F	13	8	NextSeq	4,152,008	3,290,596	G1P[8]
**F04268**	2011-2012	M	9	5	NextSeq	6,969,198	5,726,536	G3P[8]
**F05025**	2012-2013	M	11	7	NextSeq	10,218,992	8,562,184	G9P[8]
**F05040**	2012-2013	F	17	13	NextSeq	7,690,002	5,436,352	G4P[8]
**F05073**	2012-2013	F	5	2	NextSeq	13,942,494	11,052,346	G12P[8] and G2P[4]
**F05189**	2012-2013	M	38	36	NextSeq	6,809,540	5,499,944	G2P[4]
**F05209**	2012-2013	F	15	12	NextSeq	6,695,072	1,994,958	G3P[8]
**F05244**	2012-2013	M	21	17	NextSeq	5,841,152	4,651,722	G3P[8]
**F05339**	2012-2013	F	27	24	NextSeq	3,816,188	2,889,080	G2P[4]
**F05363**	2012-2013	M	26	22	NextSeq	7,743,858	6,234,022	G3P[8]
**F05466**	2012-2013	M	33	26	NextSeq	6,864,914	5,804,456	G2P[4]
**F05504**	2012-2013	M	11	6	NextSeq	4,564,188	3,549,558	G1P[8]
**F05527**	2012-2013	F	11	8	NovaSeq	14,360,232	12,150,506	G2P[4]
**F06011**	2013-2014	M	52	49	NextSeq	5,716,808	4,761,210	G2P[4]
**F06040**	2013-2014	M	20	15	NextSeq	6,174,892	5,242,310	G1P[8]
**F06047**	2013-2014	M	14	11	NextSeq	3,930,536	2,916,644	G3P[8]
**F06083**	2013-2014	F	57	54	NextSeq	6,916,620	4,956,514	G2P[4]
**F06088**	2013-2014	M	8	16	NovaSeq	22,844,448	19,708,014	G1P[8]
**F06107**	2013-2014	F	21	18	NextSeq	2,004,490	1,451,602	G9P[8]
**F06118**	2013-2014	M	7	4	NextSeq	5,449,422	4,511,916	G2P[4]
**F06184***	2013-2014	F	84	81	NextSeq	1,096,802	863,210	G12P[8]
**F07027**	2014-2015	M	19	16	NextSeq	8,856,718	6,897,804	G4P[8]
**F07049**	2014-2015	F	7	4	NextSeq	7,556,064	5,620,180	G3P[8]
**F07067**	2014-2015	Unk.	11	8	NovaSeq	7,417,542	6,367,122	G12P[8]
**F07135**	2014-2015	M	11	8	NextSeq	6,640,530	5,445,126	G3P[8]
**F07168**	2014-2015	M	25	22	NextSeq	6,726,600	5,445,596	G3P[8]
**F07215**	2014-2015	M	24	20	NextSeq	7,969,816	6,258,304	G3P[8]

**F07246**	2014-2015	M	23	20	NextSeq	6,949,114	5,640,688	G4P[8]
**F07264**	2014-2015	F	24	20	NextSeq	8,348,984	6,288,934	G12P[8]
**F07312**	2014-2015	M	15	12	NextSeq	4,228,800	2,829,952	G2P[4]
**F09155**	2016-2017	F	22	19	NextSeq	6,840,762	5,851,048	G9P[8]
**F09179**	2016-2017	F	18	14	NextSeq	7,849,094	6,342,900	G2P[4]
**F09181**	2016-2017	F	23	18	NextSeq	8,196,978	7,327,100	G2P[4]
**F09229**	2016-2017	F	26	23	NextSeq	7,656,414	6,095,436	G2P[4]
**F09256**	2016-2017	F	12	8	NextSeq	7,595,214	6,277,476	G2P[4]
**F09263**	2016-2017	M	91	88	NextSeq	5,831,356	4,518,436	G2P[4]
**F09271**	2016-2017	M	34	31	NextSeq	5,229,632	4,193,560	G9P[8]
**F09274**	2016-2017	M	24	21	NextSeq	6,071,442	4,745,648	G9P[8]
**F09317**	2016-2017	M	52	48	NextSeq	7,968,776	6,084,018	G1P[6]
**F09648**	2016-2017	M	17	14	NextSeq	6,046,962	4,949,710	G1P[8]
**F09649**	2016-2017	M	50	47	NextSeq	8,391,372	6,621,524	G9P[8]
**F09825**	2016-2017	M	55	51	NextSeq	5,481,294	4,591,514	G3P[8]
**F10090**	2017-2018	F	56	53	NextSeq	12,728,862	10,796,262	G9P[8]
**F10119**	2017-2018	M	15	11	NextSeq	11,077,134	9,557,330	G2P[4]
**F10168**	2017-2018	F	46	42	NextSeq	8,113,250	6,504,822	G3P[8]
**F10178**	2017-2018	M	9	6	NextSeq	6,628,430	5,508,922	G3P[8]
**F10181**	2017-2018	F	27	24	NextSeq	6,507,094	4,721,984	G1P[8]
**F10188**	2017-2018	M	39	36	NextSeq	7,907,220	6,323,214	G4P[8]
**F10257**	2017-2018	F	26	23	NextSeq	5,812,968	1,264,090	G3P[8]
**F10258**	2017-2018	M	40	37	NextSeq	6,798,616	5,042,866	G3P[8]
**F10324**	2017-2018	M	48	45	NextSeq	8,274,204	6,110,782	G3P[8]
			**Median (IQR)**			**Mean**		
			20 (11-26)			7,225,820	5,723,461	
						**Total**		
						737,033,630	583,793,014	

Table S2. Cq values of identified enteropathogens and internal controls by RT-qPCR assay per sample (n=92). The Cq values of the enteropathogens are color-coded according to their clinical interpretation; strong positive to weak positive transitions from dark green to light green (see below, Table S6). The given scores per pathogen are shown in brackets. IC: Internal control, PDV: Phocine distemper virus, IQR: interquartile range

Sample ID	Rotavirus	Adenovirus	Astrovirus	Enterovirus	Parechovirus	Norovirus	Sapovirus	*C. jejuni*	*C. difficile*	EAEC	EPEC	STEC	*Blasto-cystis spp.*	*D. fragilis*	*G. lamblia*	18S rRNA IC	PDV IC
**F00325**	19.24 (3)			25.03 (2)		30.11 (1)										15.35	28.87
**F00396**	22.50 (2)	20.72 (3)					31.73 (1)		30.53 (2)							22.09	31.65
**F00440**	17.90 (3)													35.14 (1)		24.92	30.57
**F00442**	26.16 (2)				33.01 (2)											31.87	32.93
**F00455**	28.00 (2)															22.88	27.96
**F00495**	21.84 (3)	32.19 (1)														19.69	29.50
**F01005**	24.16 (2)															17.74	30.05
**F01097**	14.33 (3)															19.81	31.22
**F01219**	21.85 (3)					35.39 (1)										18.84	28.81
**F01260**	21.78 (3)	32.22 (1)														24.91	30.76
**F01269**	21.59 (3)	28.93 (2)														27.20	30.78
**F01443**	19.55 (3)			28.63 (2)					31.79 (2)							25.37	28.14
**F01450**	14.34 (3)	29.58 (2)								17.73 (3)						20.91	28.47
**F02114**	16.95 (3)										26.43 (2)					26.30	32.84
**F02133**	22.37 (2)			36.07 (1)						16.76 (3)					32.42 (2)	22.14	30.74
**F02146**	19.43 (3)	27.94 (2)								35.13 (1)						18.64	28.22
**F02272**	19.98 (3)			33.83 (1)							18.45 (3)					20.41	27.84
**F02290**	20.57 (3)															20.58	31.27
**F02335**	16.54 (3)															18.50	29.36
**F02402**	22.75 (2)	23.39 (2)		28.52 (2)			28.70 (2)		28.40 (2)							20.94	29.46
**F02576**	20.17 (3)									17.60 (3)	20.58 (3)					26.62	30.63
**F02577**	23.31 (2)			31.60 (1)												19.73	30.66

**F03061**	18.98 (3)															20.98	29.89
**F03069**	22.81 (2)		26.63 (2)													21.05	31.11
**F03127**	27.70 (2)	34.24 (1)						29.05 (2)								18.23	26.34
**F03146**	21.58 (3)				34.63 (2)			22.92 (2)								26.98	26.53
**F03159**	19.71 (3)						23.45 (2)									18.98	29.10
**F03160**	19.58 (3)	21.91 (3)									34.08 (1)					20.73	30.78
**F03165**	20.92 (3)		21.95 (3)													24.10	29.53
**F03173**	18.93 (3)		26.77 (2)													16.80	30.05
**F03214**	22.47 (2)															22.11	28.60
**F03268**	19.44 (3)				32.69 (2)											23.95	28.60
**F03297**	17.93 (3)															23.42	31.13
**F03394**	24.13 (2)												33.34 (1)			21.93	31.26
**F04024**	20.79 (3)	35.54 (1)							23.49 (3)	22.57 (3)						26.50	28.41
**F04047**	19.53 (3)	27.52 (2)		34.55 (1)	34.40 (2)											22.68	29.19
**F04056**	26.01 (2)		34.38 (1)										25.14 (2)		38.27 (1)	18.50	30.20
**F04071**	23.02 (2)															24.17	30.77
**F04099**	20.33 (3)	34.96 (1)						36.13 (2)		31.82 (2)						24.70	29.54
**F04114**	25.49 (2)			33.80 (1)						16.98 (3)						20.39	31.08
**F04120**	20.04 (3)															21.28	28.91
**F04189**	25.63 (2)						32.39 (1)							35.92 (1)		23.95	29.79
**F04226**	22.43 (2)															27.60	30.46
**F04268**	19.15 (3)															23.88	28.23
**F05025**	21.14 (3)	33.64 (1)							30.06 (2)							27.40	29.20
**F05040**	28.54 (2)				24.90 (2)		27.79 (2)									26.70	31.11
**F05073**	22.40 (2)												36.30 (1)			19.97	29.41
**F05189**	22.02 (2)			33.90 (1)		31.94 (1)										22.90	29.66
**F05209**	21.23 (3)	34.49 (1)									32.91 (1)					23.97	26.55
**F05244**	28.83 (2)	34.96 (1)		23.53 (2)									36.13 (1)			23.34	30.20
**F05399**	26.61 (2)															24.19	33.15
**F05363**	19.22 (3)							31.01 (2)								23.30	29.84

**F05466**	25.67 (2)												36.03 (1)			23.50	30.87
**F05504**	21.87 (3)									18.90 (3)						24.20	30.48
**F05527**	21.15 (3)	31.10 (1)														24.95	28.76
**F06011**	23.01 (2)	33.46 (1)								17.89 (3)						24.50	31.83
**F06040**	17.02 (3)	31.32 (1)								18.12 (3)						25.27	30.38
**F06047**	24.11 (2)		36.56 (1)						32.85 (1)	22.11 (3)			7.92 (3)			18.83	29.08
**F06083**	24.32 (2)										30.45 (2)			31.55 (1)		25.84	31.53
**F06088**	18.17 (3)															19.42	26.53
**F06107**	23.83 (2)													30.84 (1)		25.12	28.27
**F06118**	21.15 (3)					36.42 (1)			25.49 (2)							22.29	26.60
**F07027**	20.63 (3)	36.03 (1)														16.33	29.41
**F07049**	28.01 (2)		27.40 (2)						30.32 (2)				35.94 (1)			24.52	28.00
**F07067**	22.87 (2)	29.85 (2)	32.54 (1)			29.93 (2)				21.81 (3)						18.11	29.44
**F07135**	21.17 (3)	31.80 (1)		35.56 (1)	23.09 (2)											26.17	28.29
**F07168**	25.38 (2)	28.17 (2)			34.77 (2)											26.15	29.71
**F07215**	21.48 (3)	27.32 (2)														21.61	28.31
**F07246**	20.68 (3)	32.36 (1)											35.63 (1)			21.55	24.51
**F07264**	22.44 (2)	33.76 (1)		25.37 (2)		33.23 (1)										25.65	30.31
**F07312**	22.35 (2)		28.89 (2)													20.77	28.40
**F09155**	27.35 (2)			34.99 (1)												25.58	31.05
**F09179**	23.03 (2)														32.53 (2)	22.26	28.53
**F09181**	20.81 (3)			32.91 (1)							21.35 (3)			31.73 (1)		25.05	30.76
**F09229**	29.06 (2)															28.26	34.81
**F09256**	27.30 (2)	29.42 (2)														25.27	29.24
**F09263**	27.36 (2)										24.25 (2)		22.96 (2)			22.68	29.58
**F09271**	20.17 (3)						26.06 (2)									24.50	29.79
**F09274**	25.21 (2)															23.47	28.79
**F09317**	23.93 (2)														32.34 (2)	26.14	29.34
**F09648**	17.41 (3)						33.77 (1)									23.04	29.02

**F09649**	26.68 (2)															22.18	31.19
**F09825**	23.14 (2)															27.85	29.64
**F10090**	18.85 (3)	32.90 (1)										34.25 (1)				25.17	30.01
**F10119**	18.65 (3)				31.38 (2)					24.15 (2)						17.62	28.52
**F10168**	17.85 (3)		30.99 (1)			31.96 (1)										24.27	25.67
**F10178**	20.69 (3)	34.38 (1)				35.37 (1)					24.61 (2)					23.51	29.31
**F10181**	17.03 (3)															21.36	29.10
**F10188**	23.34 (2)													34.21 (1)		19.27	29.68
**F10257**	20.30 (3)	30.98 (1)														25.92	30.70
**F10258**	16.65 (3)													28.00 (2)		27.11	30.76
**F10324**	24.23 (2)													30.26 (1)		24.46	31.42
																Median (IQR)	
																23 (21-25)	30 (29-31)

Table S3. Demographics of the patients harbouring the corresponding pathogens according to RT-qPCR data

Characteristics	Rotavirus	Adenovirus	Picornavirus	EAEC	Astrovirus	EPEC	*Blastocystis* spp.	Norovirus	*C. difficile*	*D. fragilis*	Sapovirus	*C. jejuni*	*G. lamblia*	STEC
**Total (%)**	92 (100)	29 (32)	20 (22)	13 (14)	9 (10)	9 (10)	9 (10)	8 (9)	8 (9)	8 (9)	7 (8)	4 (4)	4 (4)	1 (1)
**Sex**														
**Female (%)**	36 (39)	10 (34)	10 (50)	1 (8)	3 (33)	4 (44)	4 (44)	3 (38)	3 (38)	4 (50)	3 (43)	1 (25)	3 (75)	1
**Male (%)**	55 (60)	18 (62)	10 (50)	11 (85)	5 (56)	5 (56)	5 (56)	4 (50)	5 (62)	4 (50)	4 (57)	3 (75)	1 (25)	
**Unspecified (%)**	1 (1)	1 (3)		1 (8)				1 (12)						
**Age (m, median (SD))**	20 (15)	16 (11)	20 (7)	15 (15)	13 (13)	20 (27)	23 (26)	24 (15)	9 (4)	31 (16)	17 (9)	13 (8)	31 (14)	56

Table S4. Enteropathogen prevalence. Panel A shows the prevalence of enteropathogens by both RT-qPCR and NGS data. Panel B shows the prevalence of multiple co-infections according to RT-qPCR data. Rotavirus is not shown as it was present in all samples.

**A. Enteropathogen**	**RT-qPCR (n=92)**	**NGS (n=102)**
Rotavirus	100%	83%
Adenovirus	32%	5%
Enterovirus	15%	5%
EAEC	14%	
Astrovirus	10%	7%
EPEC	10%	
Blastocystis spp.	10%	
Parechovirus	9%	2%
Norovirus	9%	
C. difficile	9%	
D. fragilis	9%	
Sapovirus	8%	1%
C. jejuni	4%	
G. lamblia	4%	
STEC	1%	
Aichivirus		1%
Cardiovirus		1%
Rhinovirus		1%

**B. Co-infections**	**Prevalence**
AdV + EAEC	4%
NoV + EV	2%
AdV + EPEC	2%
AdV + EV + PeV	2%
AdV + PeV	1%
EV + EAEC	1%
PeV + EAEC	1%
HAstV + NoV	1%
AdV + *C.jejuni*	1%
AdV + *C. difficile*	1%
AdV + STEC	1%
AdV + *Blastocystis*	1%
NoV + *C .difficile*	1%
SaV + *D. fragilis*	1%
EV + *C.difficile*	1%
PeV + *C.jejuni*	1%
EV + EPEC	1%
EAEC + EPEC	1%
EPEC + *Blastocystis*	1%
EPEC + *D. fragilis*	1%
AdV + NoV + EV	1%
AdV + SaV + *C.difficile*	1%
AdV + NoV + EPEC	1%
AdV + EV + *Blastocystis*	1%
AdV + *C.difficile* + EAEC	1%
AdV + *C. jejuni* + EAEC	1%
HAstV + *C. difficile* + *Blastocystis*	1%
HAstV + *Blastocystis* + *G. lamblia*	1%
EV + EAEC + *G. lamblia*	1%
EV + EPEC + *D. fragilis*	1%
AdV + HAstV + NoV + EAEC	1%
AdV + SaV + EV + *C.difficile*	1%
HAstV + *Blastocystis* + *C.difficile* + EAEC	1%

Table S5. Discrepancies between NGS and RT-qPCR detection methods. The first category shows samples (and the corresponding Cq value) in which a viral pathogen was only detected using RT-qPCR. The Cq values are color-coded according to the clinical interpretation; strong positive to weak positive transitions from dark green to light green (see below, Table S6). The 2nd and 3rd categories indicate samples (and their raw read counts) in which only NGS analyses detected a viral pathogen. ‘Exptected’ indicates that the pathogen(s) was not included in the RT-qPCR panel, and ‘not expected’ indicates that this pathogen would be expected to be also picked up by the RT-qPCR assay.

Categories	Sample	Adenovirus	Astrovirus	Enterovirus	Parechovirus	Norovirus	Sapovirus	Other Picornavirus	Rotavirus
Only qPCR detection (NGS read counts < 100 excluded)	F00325			25.03		30.11 (GI)			
	F00396						31.73, 36.26		
	F00442				33.01				
	F00495	32.19							
	F01219					35.39 (GI)			
	F01260	32.22							
	F01269	28.93							
	F01450	29.58							
	F02133			36.07					
	F02146	27.94							
	F02272			33.83					
	F02402						28.70, 34.45		
	F03127	34.24							
	F03146				34.63				
	F03165		21.95						
	F03268				32.69				
	F04024	35.54							
	F04047	27.52		34.55	34.4				
	F04056			34.38					
	F04099	34.96							
	F04114			33.8					
	F04189						33.09		
	F05025	33.64							
	F05040						27.79		28.61
	F05189			33.9		31.94 (GII)			
	F05209	34.49							
	F05244	34.96							
	F06011	33.46							
	F06040	31.32							
	F06047		36.5						
	F06118					36.42 (GII)			
	F07027	36.03							
	F07067	29.85	32.54			29.93 (GII)			
	F07135	31.8		35.56					
	F07168	28.17			34.77				

	F07215	27.32							
	F07246	32.36							
	F07264	33.76		25.37		33.23 (GII)			
	F07312		28.89						
	F09155			34.99					
	F09179								23.85
	F09181			32.91					
	F09271						26.06		
	F09648						33.77		
	F10090	32.9							
	F10119				31.38				
	F10168					31.96 (GI)			
	F10178	34.38				35.37 (GII)			
	F10257	30.98							
**Only NGS detection (expected)**	F01005							2115676 (AiV1)	
	F05504							44978 (SAFV3)	
	F02114		31073 (VA2)						
	F02402		944964 (MLB1)						
	F02577		322 (MLB1)						
**Only NGS detection (not expected)**	F01097		8493 (HAstV1)						

Table S6. Relationship between the Cq values and the clinical interpretation for the viruses (a, d), bacteria (b, d) and parasites (c) detected by RT-qPCR

**a. Cq value**	**Clinical interpretation**	**Score**
<= 22	Strong positive	3
> 22 and <= 30	Positive	2
> 30 and < 40	Weak positive	1
>= 40	Not detectable	

**b. Cq value**	**Clinical interpretation**	**Score**
<= 24	Strong positive	3
> 24 and <= 32	Positive	2
> 32 and < 40	Weak positive	1
>= 40	Not detectable	

**c. Cq value***	**Clinical interpretation**	**Score**
< 20[a]	Strong positive	3
> 20[a] and <= 30[b]	Positive	2
> 30[b] and < 40	Weak positive	1
>= 40	Not detectable	

**d. Cq value†**	**Clinical interpretation**	**Score**
< 40	Positive	2
>= 40	Not detectable	

*For *G. lamblia*, [a]25 and [b]35
†For Parechovirus and *C. difficile*

VP4

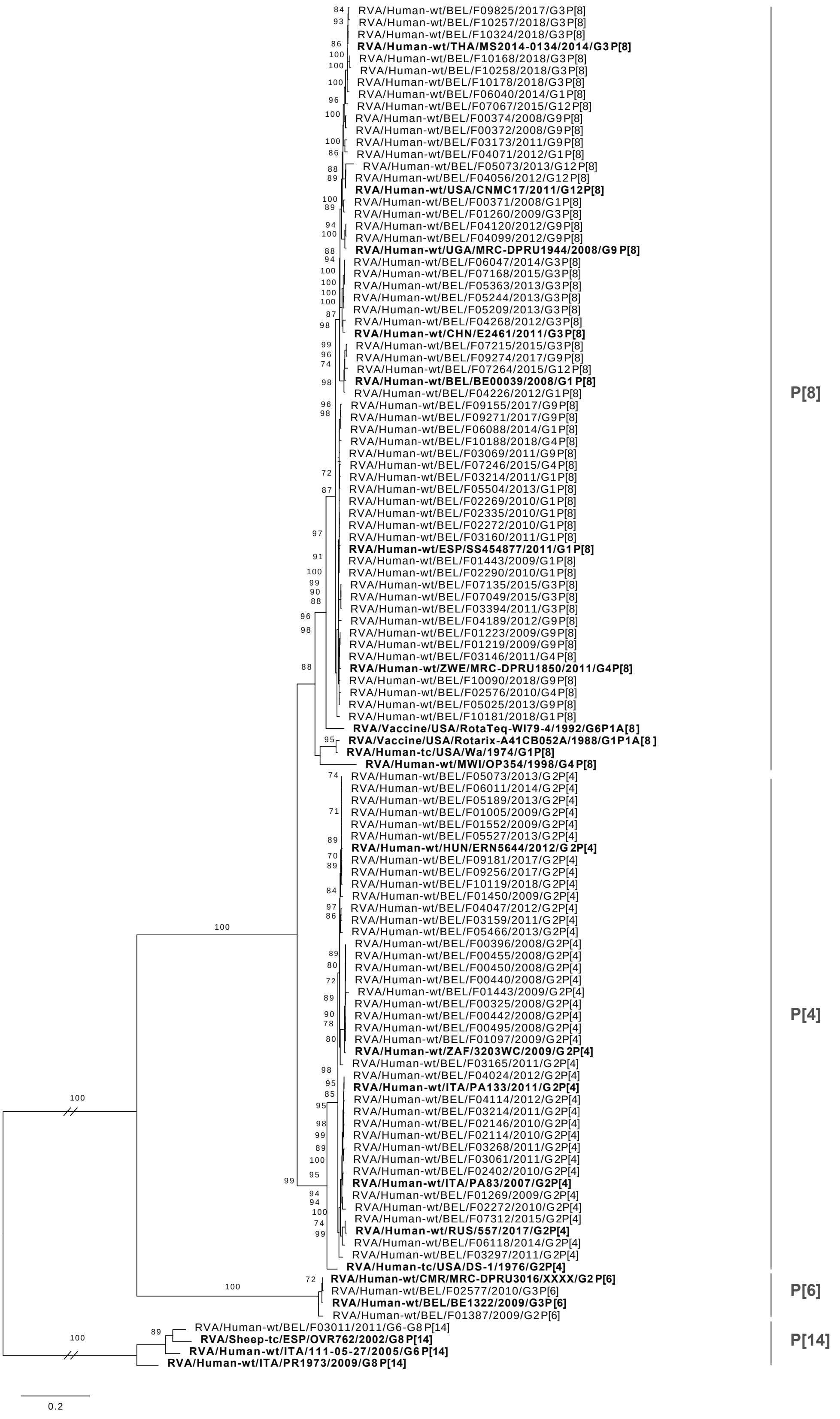

Figure S1. The maximum likelihood tree of the VP4 gene of the detected rotavirus sequences with reference rotavirus genomes. The reference genomes are marked in bold. The genotypes and lineages are shown next to the branches. Only the bootstrap values above 70% are shown. The branch lengths are drawn to scale and represent the nucleotide substitutions per site.

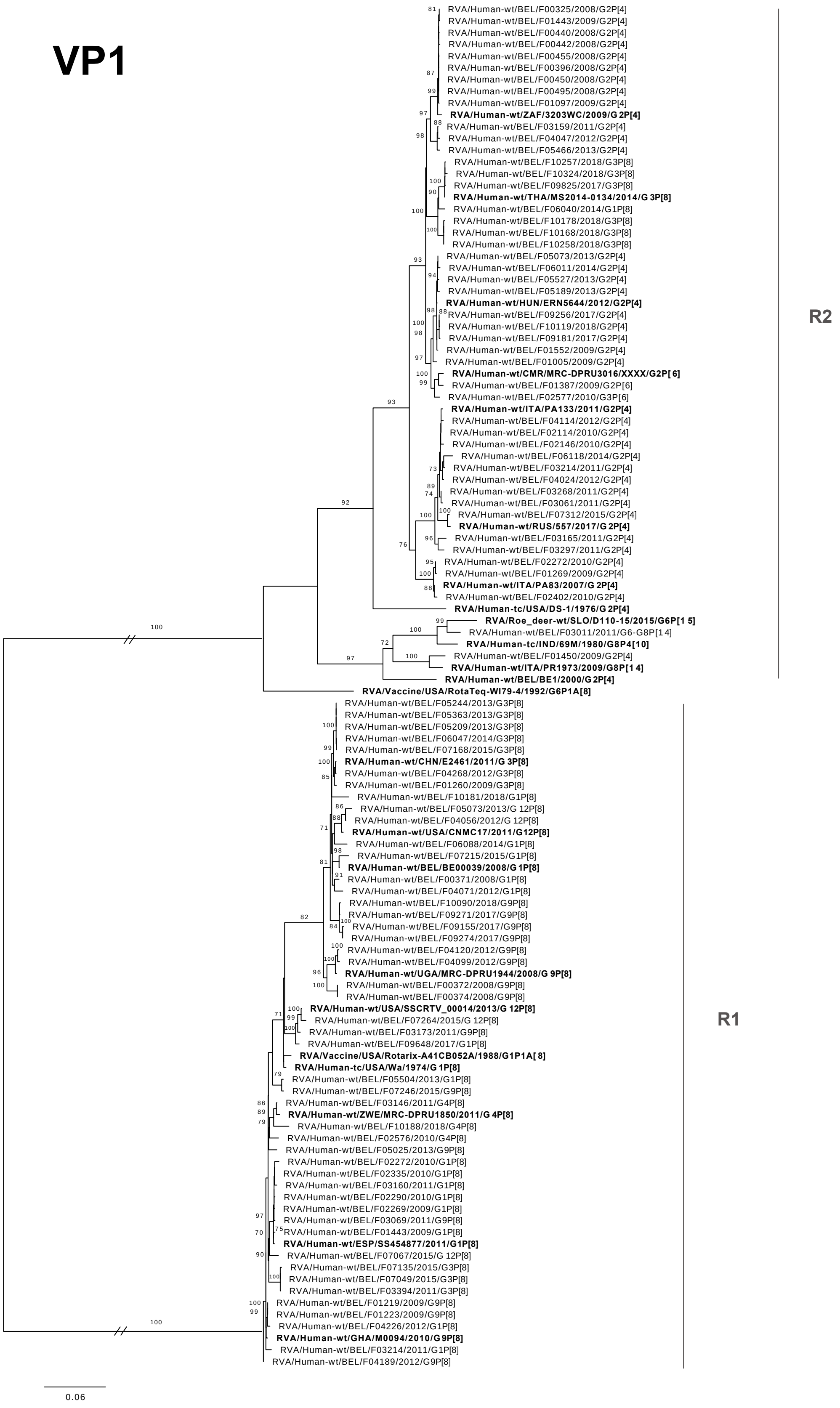

Figure S2. The maximum likelihood tree of the VP1 gene of the detected rotavirus sequences with reference rotavirus genomes. The reference genomes are marked in bold. The genotypes and lineages are shown next to the branches. Only the bootstrap values above 70% are shown. The branch lengths are drawn to scale and represent the nucleotide substitutions per site.

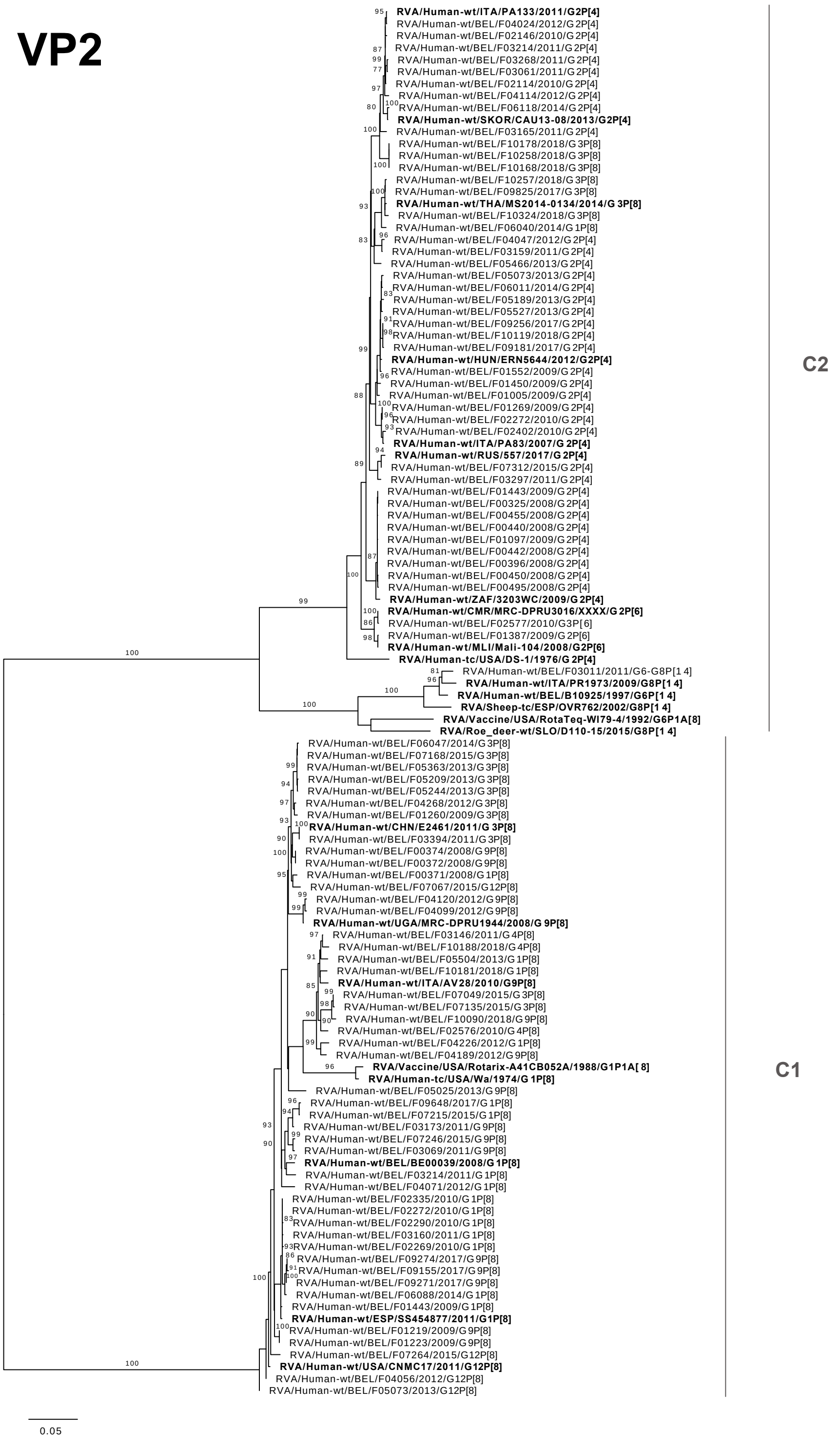

Figure S3. The maximum likelihood tree of the VP2 gene of the detected rotavirus sequences with reference rotavirus genomes. The reference genomes are marked in bold. The genotypes and lineages are shown next to the branches. Only the bootstrap values above 70% are shown. The branch lengths are drawn to scale and represent the nucleotide substitutions per site.

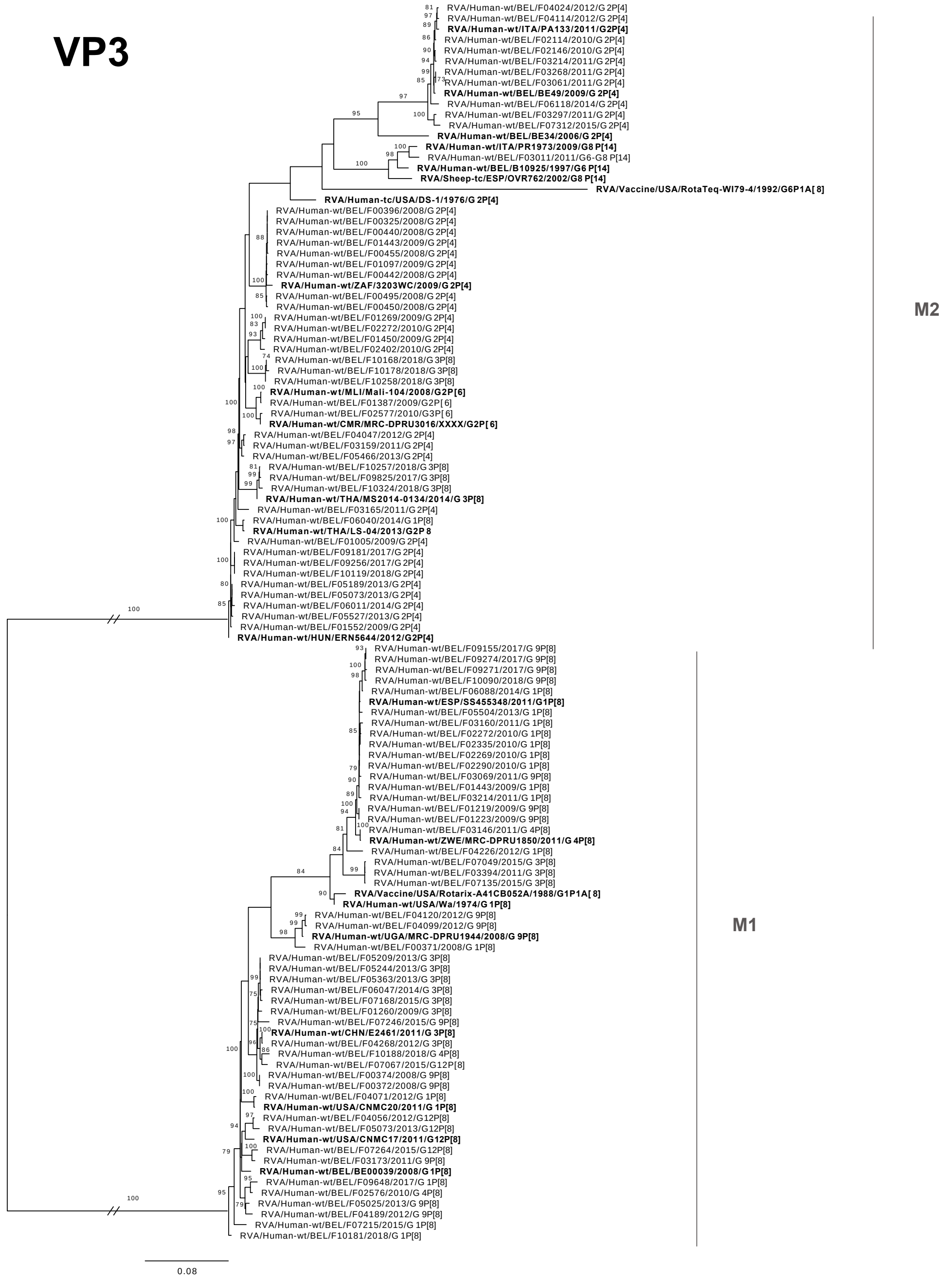

Figure S4. The maximum likelihood tree of the VP3 gene of the detected rotavirus sequences with reference rotavirus genomes. The reference genomes are marked in bold. The genotypes and lineages are shown next to the branches. Only the bootstrap values above 70% are shown. The branch lengths are drawn to scale and represent the nucleotide substitutions per site.

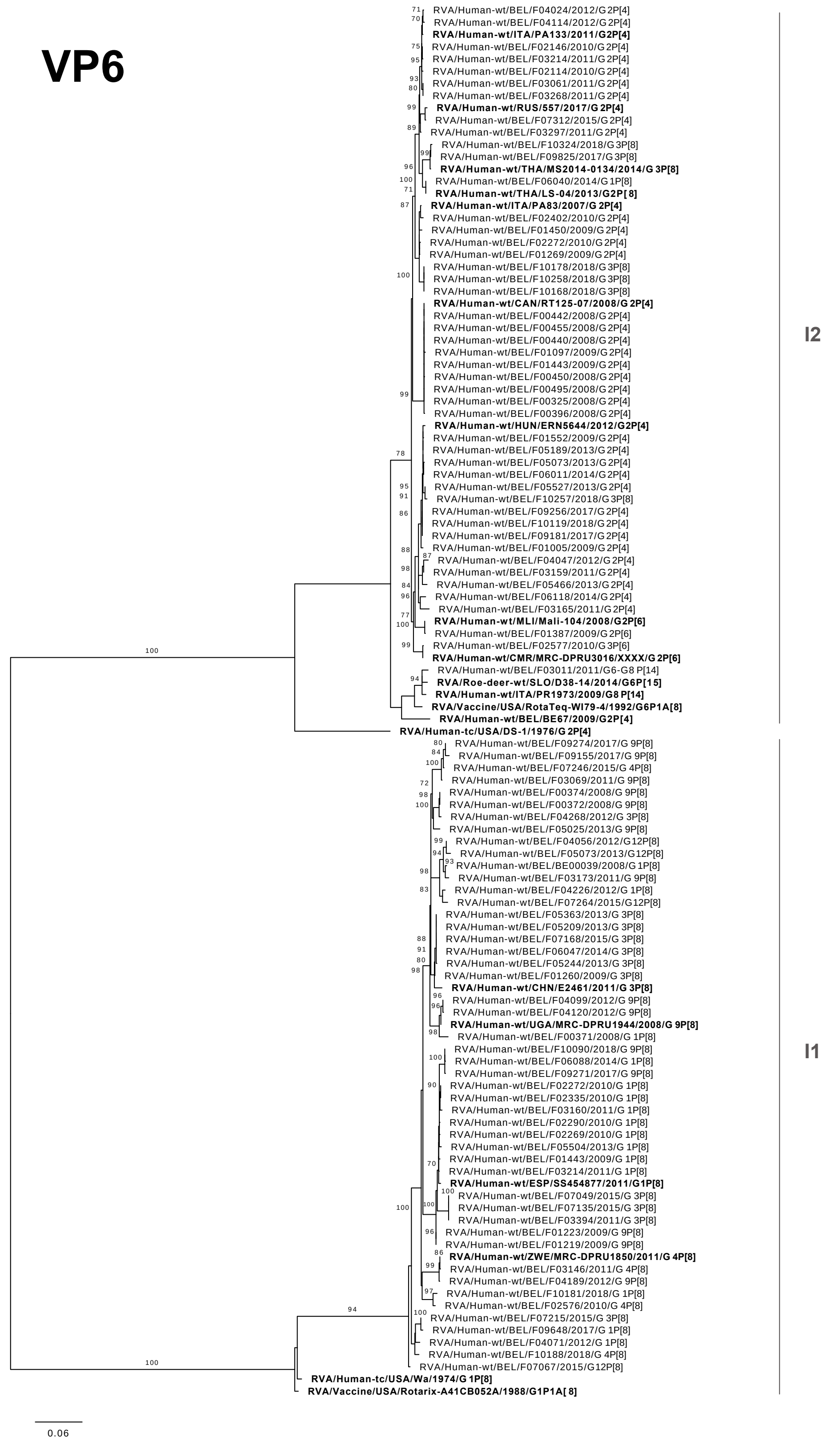

Figure S5. The maximum likelihood tree of the VP6 gene of the detected rotavirus sequences with reference rotavirus genomes. The reference genomes are marked in bold. The genotypes and lineages are shown next to the branches. Only the bootstrap values above 70% are shown. The branch lengths are drawn to scale and represent the nucleotide substitutions per site.

NSP1

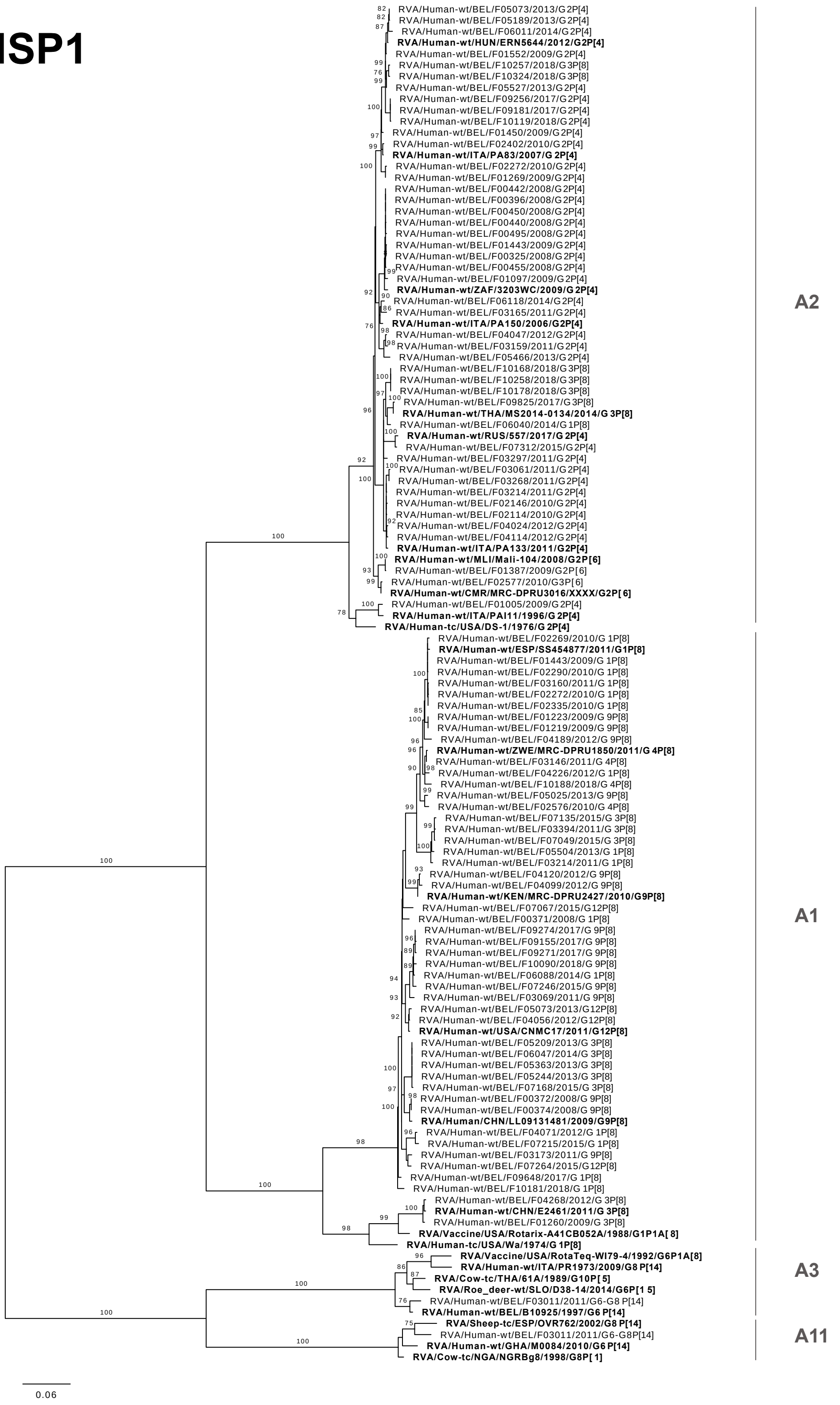

Figure S6. The maximum likelihood tree of the NSP1 gene of the detected rotavirus sequences with reference rotavirus genomes. The reference genomes are marked in bold. The genotypes and lineages are shown next to the branches. Only the bootstrap values above 70% are shown. The branch lengths are drawn to scale and represent the nucleotide substitutions per site.

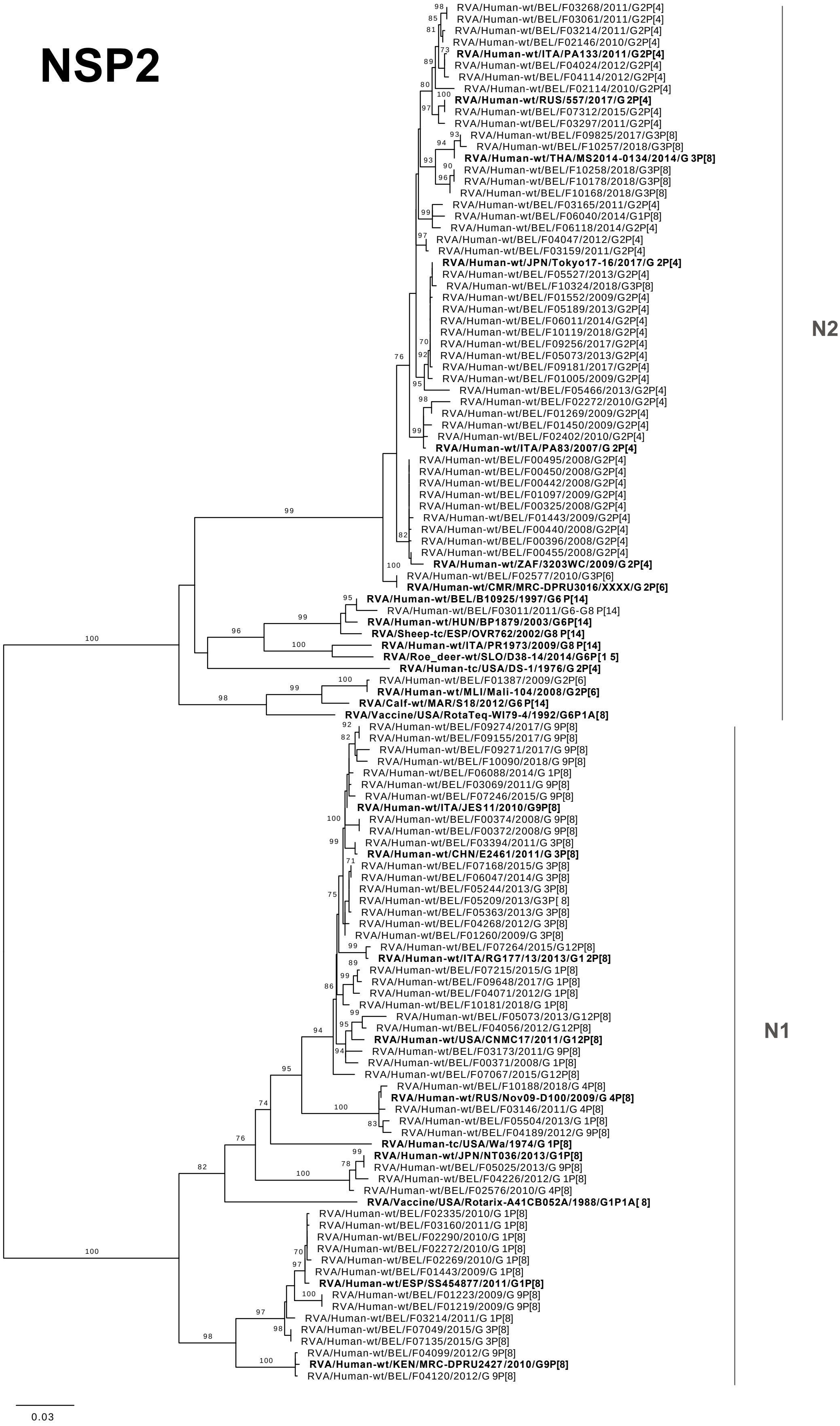

Figure S7. The maximum likelihood tree of the NSP2 gene of the detected rotavirus sequences with reference rotavirus genomes. The reference genomes are marked in bold. The genotypes and lineages are shown next to the branches. Only the bootstrap values above 70% are shown. The branch lengths are drawn to scale and represent the nucleotide substitutions per site.

NSP3

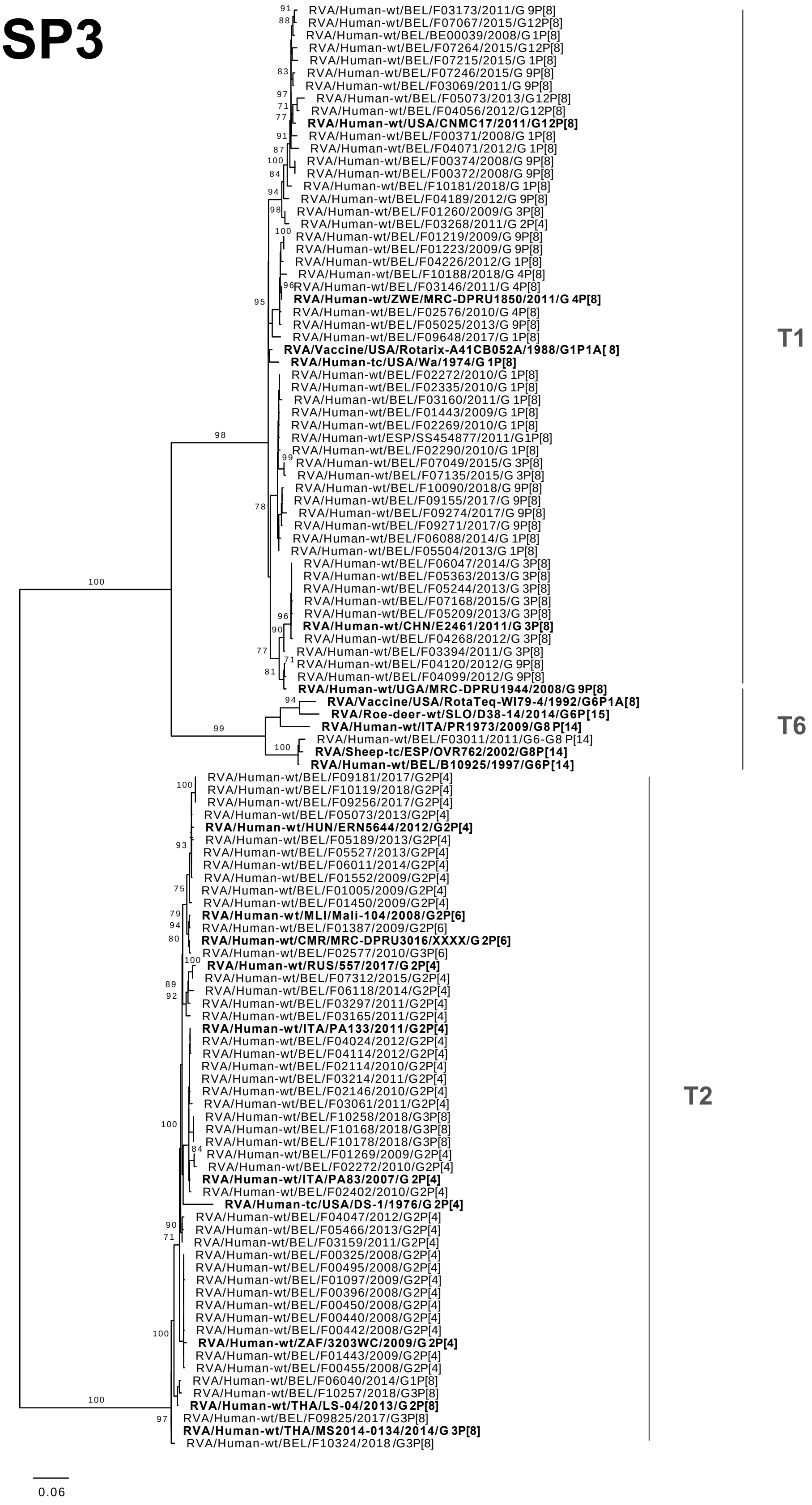

Figure S8. The maximum likelihood tree of the NSP3 gene of the detected rotavirus sequences with reference rotavirus genomes. The reference genomes are marked in bold. The genotypes and lineages are shown next to the branches. Only the bootstrap values above 70% are shown. The branch lengths are drawn to scale and represent the nucleotide substitutions per site.

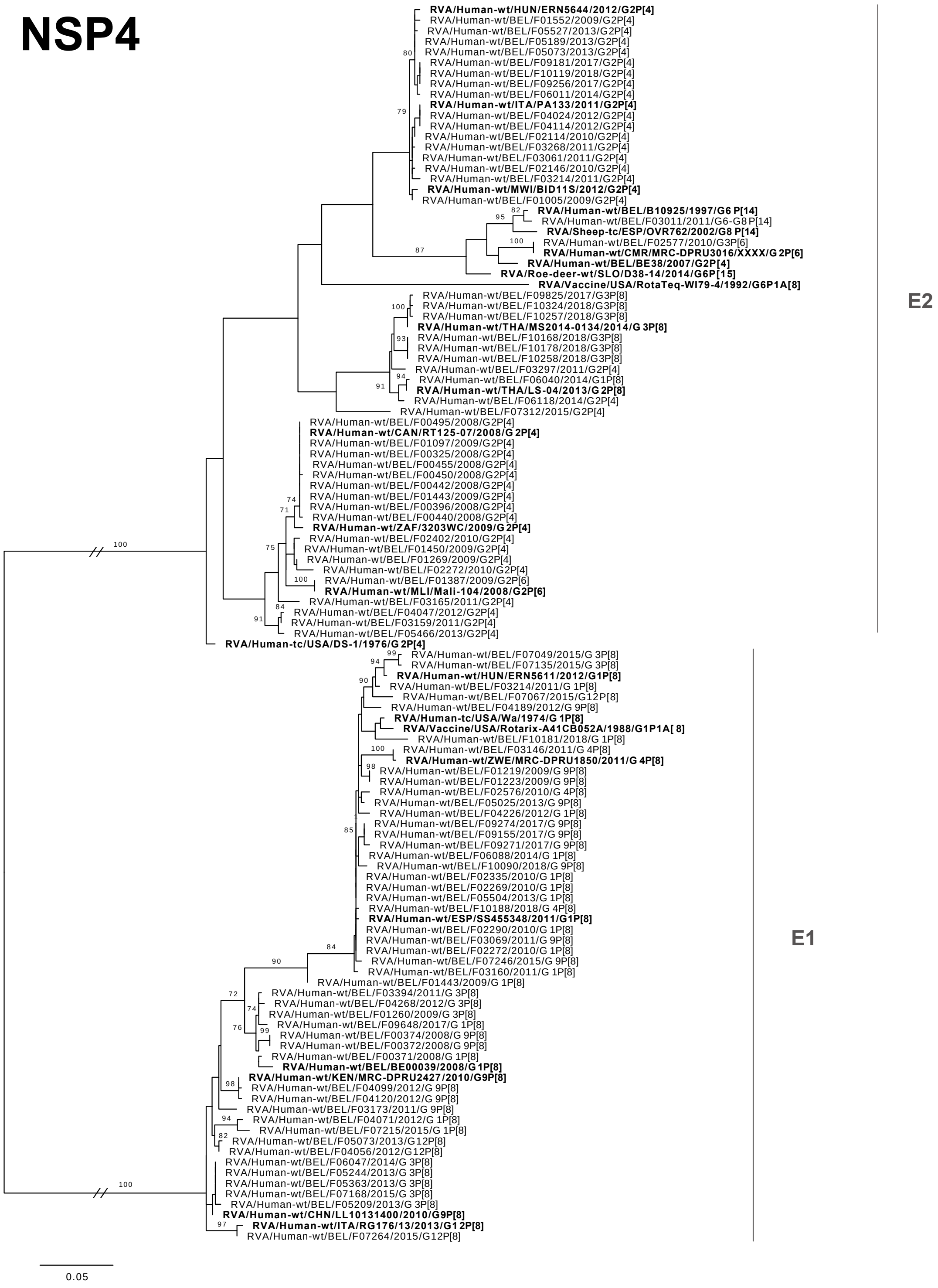

Figure S9. The maximum likelihood tree of the NSP4 gene of the detected rotavirus sequences with reference rotavirus genomes. The reference genomes are marked in bold. The genotypes and lineages are shown next to the branches. Only the bootstrap values above 70% are shown. The branch lengths are drawn to scale and represent the nucleotide substitutions per site.

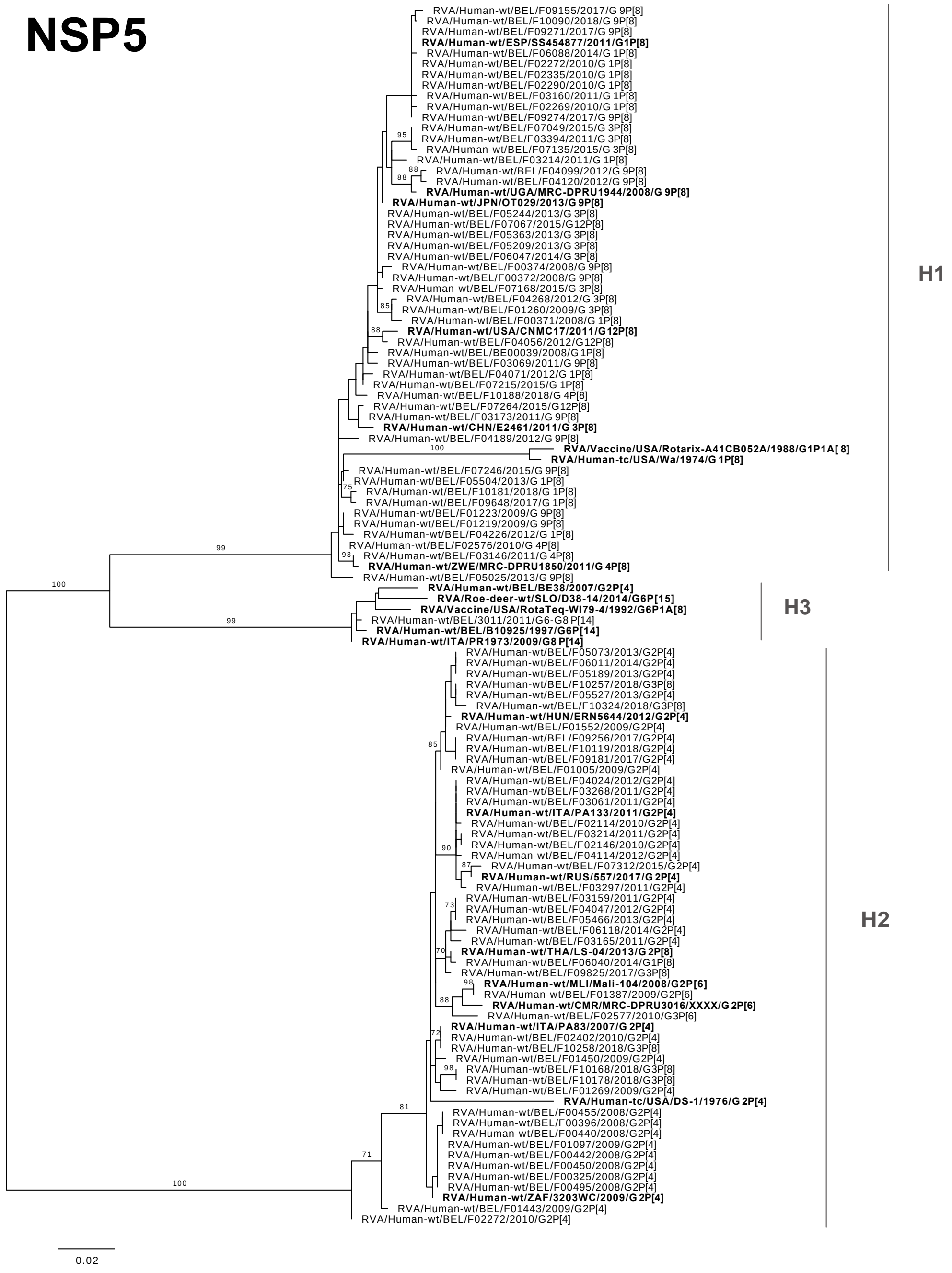

Figure S10. The maximum likelihood tree of the NSP5 gene of the detected rotavirus sequences with reference rotavirus genomes. The reference genomes are marked in bold. The genotypes and lineages are shown next to the branches. Only the bootstrap values above 70% are shown. The branch lengths are drawn to scale and represent the nucleotide substitutions per site.
