# Supplementary material for: High Prevalence of Coinfecting Enteropathogens in Suspected Rotavirus Vaccine Breakthrough Cases
Source: J Clin Microbiol. 2021 Nov 18;59(12):e01236-21. doi: 10.1128/JCM.01236-21 (PMC8601229; doi:10.1128/JCM.01236-21)
Supplement: Supplemental file 3 — Supplemental materials and methods. Download jcm.01236-21-s0003.pdf, PDF file, 0.05 MB [file jcm.01236-21-s0003.pdf]

Supplementary Materials&Methods

Before the implementation as a diagnostic test, the qPCR efficiency of all 48 targets were studied based on 12 different gBlocks Gene fragments (Integrated DNA Technologies), containing all the target sequences. 10-fold dilution series (10^{-4}, 10^{-5}, 10^{-6}, 10^{-7}, 10^{-8}) of each gBlock gene fragment enabled to assess the analytical performance of the qPCR assays.

Sensitivity and specificity analysis was based on the reference samples obtained from several sources:

- RIVM (Rijksinstituut voor Volksgezondheid en Milieu: National Institute for Public Health and the Environment):
  - Astrovirus (type 1-8), sapovirus (type I.1, I.2, II.1, II.2 and IV.1) and norovirus (type II.17)

- ATCC-culture collection samples containing lyophilised genomic DNA of *Blastocystis hominis*, *Cryptosporidium parvum*, *Giardia lamblia*, *Entamoeba histolytica* or synthetic RNA for norovirus type I

- QCMD (Quality Control for Molecular Diagnostics) quality control samples (norovirus type II, norovirus type I)

- Amplirun (Vircell) DNA controls for STEC, ETEC, EAEC, EPEC and EIEC

- UZ Leuven: Astrovirus cultures

- UZ Antwerpen: *Schistosoma*-positive samples

- UZ Brussels, reference laboratory: Cultures of all types of EPEC
